# Quantum Error-Correcting Codes Based on Orthogonal Arrays

**DOI:** 10.3390/e25040680

**Published:** 2023-04-19

**Authors:** Rong Yan, Shanqi Pang, Mengqian Chen, Fuyuan Yang

**Affiliations:** College of Mathematics and Information Science, Henan Normal University, Xinxiang 453007, China

**Keywords:** quantum error-correcting code, orthogonal array, orthogonal partition, uniform state

## Abstract

In this paper, by using the Hamming distance, we establish a relation between quantum error-correcting codes ${\left(\left(N,K,d+1\right)\right)}_{s}$ and orthogonal arrays with orthogonal partitions. Therefore, this is a generalization of the relation between quantum error-correcting codes ${\left(\left(N,1,d+1\right)\right)}_{s}$ and irredundant orthogonal arrays. This relation is used for the construction of pure quantum error-correcting codes. As applications of this method, numerous infinite families of optimal quantum codes can be constructed explicitly such as ${\left(\left(3,s,2\right)\right)}_{s}$ for all $s_{i}\geq 3$, ${\left(\left(4,s^{2},2\right)\right)}_{s}$ for all $s_{i}\geq 5$, ${\left(\left(5,s,3\right)\right)}_{s}$ for all $s_{i}\geq 4$, ${\left(\left(6,s^{2},3\right)\right)}_{s}$ for all $s_{i}\geq 5$, ${\left(\left(7,s^{3},3\right)\right)}_{s}$ for all $s_{i}\geq 7$, ${\left(\left(8,s^{2},4\right)\right)}_{s}$ for all $s_{i}\geq 9$, ${\left(\left(9,s^{3},4\right)\right)}_{s}$ for all $s_{i}\geq 11$, ${\left(\left(9,s,5\right)\right)}_{s}$ for all $s_{i}\geq 9$, ${\left(\left(10,s^{2},5\right)\right)}_{s}$ for all $s_{i}\geq 11$, ${\left(\left(11,s,6\right)\right)}_{s}$ for all $s_{i}\geq 11$, and ${\left(\left(12,s^{2},6\right)\right)}_{s}$ for all $s_{i}\geq 13$, where $s=s_{1}\cdots s_{n}$ and $s_{1},\ldots ,s_{n}$ are all prime powers. The advantages of our approach over existing methods lie in the facts that these results are not just existence results, but constructive results, the codes constructed are pure, and each basis state of these codes has far less terms. Moreover, the above method developed can be extended to construction of quantum error-correcting codes over mixed alphabets.

## 1. Introduction

As in the classical transmission of data, in the transmission of quantum information, errors are inevitable. Quantum error-correcting codes (QECCs) are designed for correcting errors in the quantum communication channels. In 1995, Shor [1] gave the first ${\left[\left[9,1,3\right]\right]}_{2}$ QECC, which was improved to the optimal ${\left[\left[5,1,3\right]\right]}_{2}$ QECC soon after [2]. In 1996, Steane revealed the natural link between basic quantum theory and linear error correcting codes of classical information theory [3]. These fundamental studies promote the rapid development of the theory of QECC. Now, QECCs have found wide applications in fault-tolerant quantum computation [4,5], quantum key distributions [6,7], and entanglement purification [8,9,10,11], etc. The construction of good QECCs has become one of the most important tasks in quantum coding theory [2,12,13,14].

The stabilizer codes are an important family of QECCs. They are studied by many researchers and a lot of results can be obtained [15,16,17,18,19,20]. Especially, based on classical Euclidean or Hermitian self-orthogonal codes, many new optimal QECCs are given [15,19,20]. Based on the coding clique, some binary QECCs, additive or non-additive, can be obtained by the graphical approach [21,22]. Although this method can be applied to the construction of non-binary QECCs ${\left(\left(N,K,d\right)\right)}_{s}$ [23] and even the QECCs ${\left(\left(N,K,d\right)\right)}_{s_{1}s_{2}\cdots s_{N}}$ over mixed alphabets [24] (short for mixed QECCs), it is difficult to search for a coding clique for bigger *N* and *d*. Moreover, for prime power *s*, even though the existence of many *s*-ary QECCs has been proved [12,19,20,23,25,26,27], only a few families of codes can be constructed explicitly [23,24,28]. Pang et al. presented a method of explicitly constructing binary QECCs by using orthogonal arrays (OAs) from difference schemes [28] and point out the superiority of the obtained QECCs to the binary stabilizer QECCs in ref. [29]. The purpose of this paper is to explicitly construct nonbinary and mixed QECCs by using OAs.

Orthogonal array, introduced by Rao [30], plays a prominent role in the design of experiments. The connection between OAs and classical error-correcting codes is revealed in refs. [31,32,33]. In 2014, Goyeneche et al. established a link between an irredundant orthogonal array (IrOA) and a uniform state [34], which is closely related to QECCs. A relation between irredundant mixed orthogonal arrays and quantum *k*-uniform states for heterogeneous systems is investigated in refs. [35,36]. Many infinite classes of uniform states for homogeneous systems and heterogeneous systems are constructed from OAs in refs. [36,37,38]. Shi et al. give a connection between QECCs and quantum information masking and point out that if a QECC Q is pure, then any state in Q is a *k*-uniform state and vice versa [39].

In recent years, more and more new OAs, especially high strength OAs, have been presented [40,41,42,43,44]. It is these new developments in OAs and uniform states that shed light on constructing QECCs from OAs.

In this paper, by using the Hamming distance, we establish a relation between quantum error-correcting codes ${\left(\left(N,K,d+1\right)\right)}_{s}$ and orthogonal arrays with orthogonal partitions. Therefore, this is a generalization of the relation between quantum error-correcting codes ${\left(\left(N,1,d+1\right)\right)}_{s}$ and irredundant orthogonal arrays. This relation is used for the construction of pure quantum error-correcting codes. As applications of this method, numerous infinite families of optimal quantum codes can be constructed explicitly such as ${\left(\left(3,s,2\right)\right)}_{s}$ for all $s_{i}\geq 3$, ${\left(\left(4,s^{2},2\right)\right)}_{s}$ for all $s_{i}\geq 5$, ${\left(\left(5,s,3\right)\right)}_{s}$ for all $s_{i}\geq 4$, ${\left(\left(6,s^{2},3\right)\right)}_{s}$ for all $s_{i}\geq 5$, ${\left(\left(7,s^{3},3\right)\right)}_{s}$ for all $s_{i}\geq 7$, ${\left(\left(8,s^{2},4\right)\right)}_{s}$ for all $s_{i}\geq 9$, ${\left(\left(9,s^{3},4\right)\right)}_{s}$ for all $s_{i}\geq 11$, ${\left(\left(9,s,5\right)\right)}_{s}$ for all $s_{i}\geq 9$, ${\left(\left(10,s^{2},5\right)\right)}_{s}$ for all $s_{i}\geq 11$, ${\left(\left(11,s,6\right)\right)}_{s}$ for all $s_{i}\geq 11$, and ${\left(\left(12,s^{2},6\right)\right)}_{s}$ for all $s_{i}\geq 13$, where $s=s_{1}\cdots s_{n}$ and $s_{1},\ldots ,s_{n}$ are all prime powers. The advantages of our approach over existing methods lie in the facts that these results are not just existence results, but constructive results, the codes constructed are pure, and each basis state of these codes has far less terms. Moreover, the above method developed can be extended to the construction of QECCs over mixed alphabets.

This paper is organized as follows. In Section 2, we review some basic knowledge about orthogonal arrays and QECCs. In Section 3, we present a general method of constructing QECCs over a single alphabet by using OAs and construct numerous infinite families of optimal quantum codes. Afterwards, by expansive replacement of an orthogonal array, this method is extended to the construction of QECCs over mixed alphabets. In Section 4, some examples are provided. Some conclusions are drawn in Section 5. The two explicitly constructed QECCs ${\left(\left(6,5^{2},3\right)\right)}_{5}$ and ${\left(\left(7,7^{3},3\right)\right)}_{7}$ are listed in Appendix A.

## 2. Preliminaries

First, the notations used in this paper are listed as follows.

Let $A^{T}$ be the transposition of matrix *A* and $\left(s\right)={\left(0,1,\ldots ,s-1\right)}^{T}$. Let $0_{r}$ denote the r×1 vector of 0s. Let $Z_{s}^{n}$ denote the *n*- dimensional space over a ring $Z_{s}=\left\{0,1,\ldots ,s-1\right\}$. If $A={\left(a_{ij}\right)}_{n\times m}$ and $B={\left(b_{uv}\right)}_{s\times t}$ with elements from a Galois field with binary operations (+and·), the Kronecker sum A⊕B is defined as $A⊕B={\left(a_{ij}+B\right)}_{sn\times tm}$ where $a_{ij}+B$ represents the s×t matrix with entries $a_{ij}+b_{uv}$ (1≤u≤s,1≤v≤t). Let ${C^{s}}^{⊗N}={\underset{︸}{C^{s}⊗C^{s}⊗\cdots ⊗C^{s}}}_{N}$.

Some basic knowledge about OA and QECC is given.

**Definition 1**  ([37]). *An orthogonal array OA(r,N,s,t) of strength t is an r×N matrix with elements from $Z_{s}$, with the property that, in any r×t submatrix, all possible combinations of t symbols appear equally often as a row.*

**Definition 2**  ([43]). *Let A be an OA(r,N,s,t). Suppose that the rows of A can be partitioned into K submatrices $A_{1},\ldots ,A_{K}$ such that each $A_{i}$ is an $OA(r/K,N,s,t^{\prime })$ with $t^{\prime }\geq 0$. Then the set $\left\{A_{1},\ldots ,A_{K}\right\}$ is called an orthogonal partition of strength $t^{\prime }$ of A.*

**Definition 3**  ([45]). *Let $R_{1},\ldots ,R_{n}$ be the rows of an n×k matrix A with entries from $Z_{s}$ . The Hamming distance $HD(R_{u},R_{v})$ between $R_{u}=\left(a_{u1},\ldots ,a_{uk}\right)$ and $R_{v}=\left(a_{v1},\ldots ,a_{vk}\right)$ is defined as follows:*

$$HD\left(R_{u},R_{v}\right)=\left|\left\{r:1\leq r\leq k,a_{ur}\neq a_{vr}\right\}\right|.$$

*In this paper, MD(L) denotes the minimum Hamming distance between two distinct rows of an OA L.*

**Definition 4**  ([34]). *An OA(r,N,s,t) is said to be an irredundant orthogonal array if, in any r×(N−t) subarray, all of its rows are different.*

**Definition 5**  ([37]). *A pure quantum state of N subsystems with s levels is said to be d-uniform if all of its reductions to d qudits are maximally mixed.*

A link between an IrOA of strength *d* and a *d*-uniform state is established and an ${\left(\left(N,1,d+1\right)\right)}_{s}$ QECC is one-to-one connected to a *d*-uniform state of *N* qudits in ref. [34]. Hence the uniform state corresponding to an IrOA of strength *d* can be seen as an ${\left(\left(N,1,d+1\right)\right)}_{s}$ QECC.

**Lemma 1**  ([39]). *Let Q be a subspace of ${C^{s}}^{⊗N}$. If Q is an ${\left(\left(N,K,d+1\right)\right)}_{s}$ QECC, then for any d parties, the reductions of all states in Q to the d parties are identical. The converse is true. Further if Q is pure, then any state in Q is a d-uniform state. The converse is also true.*

It follows from Lemma 1 that an ${\left(\left(N,K,d+1\right)\right)}_{s}$ QECC corresponds to a special subspace of ${C^{s}}^{⊗N}$. Therefore, the lemma can also be regarded as the definition of a QECC ${\left(\left(N,K,d+1\right)\right)}_{s}$ in ref. [46], where *N* is the number of qudits, *K* is the dimension of the encoding state, d+1 is the minimum distance, and *s* is the alphabet size. A QECC can also be denoted by ${\left[\left[N,k,d+1\right]\right]}_{s}$ where $k=\log_{s}K$ usually. In this paper, we mainly use the notation ${\left(\left(N,K,d+1\right)\right)}_{s}$ because it is convenient to reveal the relation between codes ${\left(\left(N,K,d+1\right)\right)}_{s}$ and orthogonal arrays with orthogonal partitions. An ${\left(\left(N,K,d+1\right)\right)}_{s}$ QECC has the quantum Singleton bound $K\leq s^{N-2d}$. A QECC saturating the bound is called optimal.

The following are some important properties of OAs.

**Lemma 2**  ([47]). *Taking the runs in an OA(r,N,s,t) that begin with 0 (or any other particular symbol) and omitting the first column yields an OA(r/s,N−1,s,t−1). If we assume that these are the initial runs, the process can be represented by the following diagram:*
$\begin{array}{c} 0\\ \vdots \\ 0 \end{array}$OA(r/s,N−1,s,t−1)$\begin{array}{c} 1\\ \vdots \\ 1 \end{array}$OA(r/s,N−1,s,t−1)⋮⋮

**Lemma 3**  ([48]). *If s≥2 is a prime power then an $OA(s^{t},s+1,s,t)$ of index unity exists whenever s≥t−1≥0.*

**Lemma 4**  ([48]). *If $s=2^{m}$ and m≥1, then there exists an $OA(s^{3},s+2,s,3)$.*

**Lemma 5**  ([37]). *The minimal distance of an $OA(s^{t},N,s,t)$ is N−t+1 for s≥2 and t≥1.*

**Lemma 6**  ([48]). *Two $OA(r_{1},N,s_{1},t)$ and $OA(r_{2},N,s_{2},t)$ can produce an $OA(r_{1}r_{2},N,s_{1}s_{2},t)$.*

**Lemma 7.**  *Assume that A is an $OA(r_{1},N,s_{1},t)$ with $MD\left(A\right)=h_{1}$, and that B is an $OA(r_{2},N,$$s_{2},t)$ with $MD\left(B\right)=h_{2}$. Let $h=min\{h_{1},h_{2}\}$. Then, there exists an $OA(r_{1}r_{2},N,s_{1}s_{2},t)$ with MD=h.*

**Proof.**  Let

$$A=\left(\begin{array}{cccc} a_{11} & a_{12} & \ldots & a_{1N}\\ a_{21} & a_{22} & \ldots & a_{2N}\\ \vdots & \vdots & \cdots & \vdots \\ a_{r_{1}1} & a_{r_{1}2} & \ldots & a_{r_{1}N} \end{array}\right)\mathrm{and}\;\;B=\left(\begin{array}{cccc} b_{11} & b_{12} & \ldots & b_{1N}\\ b_{21} & b_{22} & \ldots & b_{2N}\\ \vdots & \vdots & \cdots & \vdots \\ b_{r_{2}1} & b_{r_{2}2} & \ldots & b_{r_{2}N} \end{array}\right).$$

The $OA(r_{1}r_{2},N,s_{1}s_{2},t)$ exists from Lemma 6. It can be written as

$$C=\left(\begin{array}{cccc} \left(a_{11},b_{11}\right) & \left(a_{12},b_{12}\right) & \cdots & \left(a_{1N},b_{1N}\right)\\ \left(a_{11},b_{21}\right) & \left(a_{12},b_{22}\right) & \cdots & \left(a_{1N},b_{2N}\right)\\ \vdots & \vdots & \cdots & \vdots \\ \left(a_{11},b_{r_{2}1}\right) & \left(a_{12},b_{r_{2}2}\right) & \cdots & \left(a_{1N},b_{r_{2}N}\right)\\ \vdots & \vdots & \ldots & \vdots \\ \left(a_{r_{1}1},b_{11}\right) & \left(a_{r_{1}2},b_{12}\right) & \cdots & \left(a_{r_{1}N},b_{1N}\right)\\ \left(a_{r_{1}1},b_{21}\right) & \left(a_{r_{1}2},b_{22}\right) & \cdots & \left(a_{r_{1}N},b_{2N}\right)\\ \vdots & \vdots & \cdots & \vdots \\ \left(a_{r_{1}1},b_{r_{2}1}\right) & \left(a_{r_{1}2},b_{r_{2}2}\right) & \cdots & \left(a_{r_{1}N},b_{r_{2}N}\right) \end{array}\right..$$

Consider MD(C). In *C*, take any two rows $c_{1}=\left(\left(a_{e1},b_{g1}\right),\left(a_{e2},b_{g2}\right),\ldots ,\left(a_{eN},b_{gN}\right)\right)$ and $c_{2}=(\left(a_{f1},b_{v1}\right),(a_{f2},$ $b_{v2}),\ldots ,\left(a_{fN},b_{vN}\right))$. Correspondingly, $a_{1}=\left(a_{e1},a_{e2},\ldots ,a_{eN}\right)$ and $a_{2}=(a_{f1},a_{f2},\ldots ,$ $a_{fN})$ are two rows of *A* while $b_{1}=\left(b_{g1},b_{g2},\ldots ,b_{gN}\right)$ and $b_{2}=\left(b_{v1},b_{v2},\ldots ,b_{vN}\right)$ are two rows of *B*. Let $H_{c_{1}c_{2}}=\left|\left\{i|a_{ei}\neq a_{fi}\;\;\mathrm{and}\;\;b_{gi}\neq b_{vi},i=1,\ldots ,N.\right\}\right|$, where |A| denotes the number of elements of a set *A*. We have

$$HD\left(c_{1},c_{2}\right)=\left\{\begin{array}{cc} HD\left(b_{1},b_{2}\right)\geq h_{2} & \mathrm{if}\;\;e=f\;\;\mathrm{and}\;\;g\neq v,\\ HD\left(a_{1},a_{2}\right)\geq h_{1} & \mathrm{if}\;\;e\neq f\;\;\mathrm{and}\;\;g=v,\\ HD\left(a_{1},a_{2}\right)+HD\left(b_{1},b_{2}\right)-H_{c_{1}c_{2}}\geq max\left\{h_{1},h_{2}\right\} & \mathrm{if}\;\;e\neq f\;\;\mathrm{and}\;\;g\neq v. \end{array}\right.$$

Therefore, MD(C)=h. □

**Lemma 8.**  *Under the conditions of Lemma 7, suppose A has an orthogonal partition $\left\{A_{1},\ldots ,A_{n}\right\}$ of strength $t^{\prime }$ with $MD\left(A_{i}\right)=h_{1}^{\prime }$ for i∈{1,…,n} and that B has an orthogonal partition $\left\{B_{1},\ldots ,B_{m}\right\}$ of strength $t^{\prime }$ with $MD\left(B_{j}\right)=h_{2}^{\prime }$ for j∈{1,…,m}. Let $h^{\prime }=min\left\{h_{1}^{\prime },h_{2}^{\prime }\right\}$. Then the $OA(r_{1}r_{2},N,s_{1}s_{2},t)$ produced by Lemma 7 has an orthogonal partition $\left\{C_{11},\ldots ,C_{nm}\right\}$ of strength $t^{\prime }$ with $MD\left(C_{ij}\right)=h^{\prime }\geq h$.*

**Proof.**  Let $C=OA(r_{1}r_{2},N,s_{1}s_{2},t)$. Denote

$$A_{i}=\left(\begin{array}{cccc} a_{11}^{i} & a_{12}^{i} & \cdots & a_{1N}^{i}\\ a_{21}^{i} & a_{22}^{i} & \cdots & a_{2N}^{i}\\ \vdots & \vdots & \cdots & \vdots \\ a_{l1}^{i} & a_{l2}^{i} & \cdots & a_{lN}^{i} \end{array}\right),\;\;B_{j}=\left(\begin{array}{cccc} b_{11}^{j} & b_{12}^{j} & \cdots & b_{1N}^{j}\\ b_{21}^{j} & b_{22}^{j} & \cdots & b_{2N}^{j}\\ \vdots & \vdots & \cdots & \vdots \\ b_{g1}^{j} & b_{g2}^{j} & \cdots & b_{gN}^{j} \end{array}\right),$$

where $l=\frac{r_{1}}{n}$, $g=\frac{r_{2}}{m}$. We define

$$C_{ij}=\left(\begin{array}{cccc} \left(a_{11}^{i},b_{11}^{j}\right) & \left(a_{12}^{i},b_{12}^{j}\right) & \cdots & \left(a_{1N}^{i},b_{1N}^{j}\right)\\ \left(a_{11}^{i},b_{21}^{j}\right) & \left(a_{12}^{i},b_{22}^{j}\right) & \cdots & \left(a_{1N}^{i},b_{2N}^{j}\right)\\ \vdots & \vdots & \cdots & \vdots \\ \left(a_{11}^{i},b_{g1}^{j}\right) & \left(a_{12}^{i},b_{g2}^{j}\right) & \cdots & \left(a_{1N}^{i},b_{gN}^{j}\right)\\ \vdots & \vdots & \cdots & \vdots \\ \left(a_{l1}^{i},b_{11}^{j}\right) & \left(a_{l2}^{i},b_{12}^{j}\right) & \cdots & \left(a_{lN}^{i},b_{1N}^{j}\right)\\ \left(a_{l1}^{i},b_{21}^{j}\right) & \left(a_{l2}^{i},b_{22}^{j}\right) & \cdots & \left(a_{lN}^{i},b_{2N}^{j}\right)\\ \vdots & \vdots & \cdots & \vdots \\ \left(a_{l1}^{i},b_{g1}^{j}\right) & \left(a_{l2}^{i},b_{g2}^{j}\right) & \cdots & \left(a_{lN}^{i},b_{gN}^{j}\right) \end{array}\right).$$

Then, $C_{ij}$ is an $OA(lg,N,s_{1}s_{2},t^{\prime })$ for i∈{1,…,n},j∈{1,…,m}. By Lemma 7, $MD\left(C_{ij}\right)=h^{\prime }$. Since $h_{1}^{\prime }\geq h_{1}$ and $h_{2}^{\prime }\geq h_{2}$, we have $h^{\prime }=min\left\{h_{1}^{\prime },h_{2}^{\prime }\right\}\geq min\left\{h_{1},h_{2}\right\}=h$. Obviously, $\left\{C_{11},\ldots ,C_{nm}\right\}$ is an orthogonal partition of *C*. □

## 3. The Construction of QECCs Based on OAs

We present a general method for constructing QECCs from OAs in this section. Theorem 1 reveals a relation between a QECC and an OA with an orthogonal partition. With Theorem 1 and the existence of $OA(s^{t},s+1,s,t)$, Theorem 2 produces the ${\left(\left(N,s^{l},t-l+1\right)\right)}_{s}$ QECCs including several infinite classes of optimal QECCs in Corollary 1. In Theorem 3, several special QECCs can be directly obtained by using Theorem 1. Two optimal QECCs ${\left(\left(6,5^{2},3\right)\right)}_{5}$ and ${\left(\left(7,7^{3},3\right)\right)}_{7}$ are presented in Theorem 4. The production construction of the obtained QECCs is given in Theorem 5. Consequently, Corollary 3 improves Theorem 2 and Corollary 1. Theorem 6 is the generalization of Theorem 2 to construction of QECCs over mixed alphabets.

Goyeneche et al. reveal the relation between a ${\left(\left(N,1,d+1\right)\right)}_{s}$ QECC and an IrOA [34], while the following result is the generalization of this relation.

**Theorem 1.**  *Assume that there exists an OA(r,N,s,t) with MD=h and an orthogonal partition $\left\{A_{1},\ldots ,A_{K}\right\}$ of strength $t^{\prime }$. Let $d=min\{t^{\prime },h-1\}$. Then, there exists an ${\left(\left(N,K,d+1\right)\right)}_{s}$ QECC.*

**Proof.**  By Definition 4, the OA(r,N,s,t) and $A_{i}\;\left(i=1,\ldots ,K\right)$ are an IrOA(r,N,s,d) and an IrOA$(\frac{r}{K},$ N,s,d), respectively. From the link between IrOAs and uniform states in ref. [34] and $\left\{A_{1},\ldots ,A_{K}\right\}$, we can obtain *K d*-uniform states $\{|\varphi_{1}\rangle ,\ldots ,|\varphi_{K}\rangle \}$, which can be used as an orthogonal basis. By Lemma 1, the complex subspace spanned by the orthogonal basis is an ${\left(\left(N,K,d+1\right)\right)}_{s}$ QECC. □

**Remark 1.**  *Using Theorem 1, one can easily obtained a QECC because of the one-to-one correspondence between the orthogonal basis $\{|\varphi_{1}\rangle ,\ldots ,|\varphi_{K}\rangle \}$ of the code and the orthogonal partition $\left\{A_{1},\ldots ,A_{K}\right\}$ of an orthogonal array. The codes from Theorem 1 are better than the ones in ref. [23] in terms of the number of the terms of basis states of codes. The number is decreasing geometrically. For example, the OA$\left(9,3,3,2\right)={\left(\begin{array}{ccccccccc} 0 & 1 & 2 & 0 & 1 & 2 & 0 & 1 & 2\\ 0 & 1 & 2 & 1 & 2 & 0 & 2 & 0 & 1\\ 0 & 1 & 2 & 2 & 0 & 1 & 1 & 2 & 0 \end{array}\right)}^{T}$ with minimal distance 2 has the partition $\left\{A_{1},A_{2},A_{3}\right\}$ of strength 1, where $A_{1}=\left(\begin{array}{ccc} 0 & 0 & 0\\ 1 & 1 & 1\\ 2 & 2 & 2 \end{array}\right)$, $A_{2}=\left(\begin{array}{ccc} 0 & 1 & 2\\ 1 & 2 & 1\\ 2 & 0 & 1 \end{array}\right)$ and $A_{3}=\left(\begin{array}{ccc} 0 & 2 & 1\\ 1 & 0 & 2\\ 2 & 1 & 0 \end{array}\right)$. Every row of $A_{i}$ is put in kets and summed to produce 1-uniform state $|\varphi_{i}\rangle$, i.e., $|\varphi_{1}\rangle \;=|000\rangle +|111\rangle +|222\rangle$, $|\varphi_{2}\rangle \;=|012\rangle +|120\rangle +|201\rangle$ and $|\varphi_{3}\rangle \;=|021\rangle +|102\rangle +|210\rangle$. A ${\left(\left(3,3,2\right)\right)}_{3}$ QECC can be obtained easily, and its three basis states are $|\varphi_{i}\rangle$ for i=1,2,3. The number of the terms of each basis state of this code is 3. Base on the coding group {(0,0,0),(1,0,2),(2,0,1)}, another ${\left(\left(3,3,2\right)\right)}_{3}$ QECC can be obtained in ref. [23] and the three graph-state bases are $|\psi_{1}\rangle \;=|000\rangle +|001\rangle +|002\rangle +|010\rangle +\omega |011\rangle +\omega^{2}|012\rangle +|020\rangle +\omega^{2}|021\rangle +\omega |022\rangle +|100\rangle +|101\rangle +|102\rangle +\omega |110\rangle +\omega^{2}|111\rangle +|112\rangle +\omega^{2}|120\rangle +\omega |121\rangle +|122\rangle +|200\rangle +|201\rangle +|202\rangle +\omega^{2}|210\rangle +|211\rangle +\omega |212\rangle +\omega |220\rangle +|221\rangle +\omega^{2}|222\rangle$, $|\psi_{2}\rangle \;=|000\rangle +\omega^{2}|001\rangle +\omega |002\rangle +|010\rangle +|011\rangle +|012\rangle +|020\rangle +\omega |021\rangle +\omega^{2}|022\rangle +\omega |100\rangle +|101\rangle +\omega^{2}|102\rangle +\omega^{2}|110\rangle +\omega^{2}|111\rangle +\omega^{2}|112\rangle +|120\rangle +\omega |121\rangle +\omega^{2}|122\rangle +\omega^{2}|200\rangle +\omega |201\rangle +|202\rangle +\omega |210\rangle +\omega |211\rangle +\omega |212\rangle +|220\rangle +\omega |221\rangle +\omega^{2}|222\rangle$, $|\psi_{3}\rangle \;=|000\rangle +\omega |001\rangle +\omega^{2}|002\rangle +|010\rangle +\omega^{2}|011\rangle +\omega |012\rangle +|020\rangle +|021\rangle +|022\rangle +\omega^{2}|100\rangle +|101\rangle +\omega |102\rangle +|110\rangle +\omega^{2}|111\rangle +\omega |112\rangle +\omega |120\rangle +\omega |121\rangle +\omega |122\rangle +\omega |200\rangle +\omega^{2}|201\rangle +|202\rangle +|210\rangle +\omega^{2}|211\rangle +\omega |212\rangle +\omega^{2}|220\rangle +\omega^{2}|221\rangle +\omega^{2}|222\rangle$, where $\omega =e^{i\frac{2\pi }{3}}$. Obviously, the number of the terms of every basis state of this code in ref. [23] is 27.*

**Theorem 2.**  *For a prime power s and integers N,l,t, if 2t≤l+N≤s+1 and t≥l≥1, then there exists an ${\left(\left(N,s^{l},t-l+1\right)\right)}_{s}$ QECC. Moreover, the QECC is optimal if and only if l+N=2t.*

**Proof.**  Since s+1≥2t, we have s≥2t−1. So s≥t−1. By Lemma 3, an $OA(s^{t},s+1,s,t)$ exists. Then there exists an $OA(s^{t},l+N,s,t)$ if l+N≤s+1.

After permutation of rows, the $OA(s^{t},l+N,s,t)$ has the following form

$$L=(\left(s\right)⊕0_{s^{t-1}},0_{s}⊕\left(s\right)⊕0_{s^{t-2}},\ldots ,0_{s^{l-1}}⊕\left(s\right)⊕0_{s^{t-l}},V)\;\;\;\;\;\;\;\;\;\;\;\;\;\;\;\;\;\;\;\;\;\;\;\;\;\;\;\;\;\;\;\;\;\;\;\;\;$$

$$=\left(\left(s\right)⊕0_{s^{t-1}},0_{s}⊕\left(s\right)⊕0_{s^{t-2}},\ldots ,0_{s^{l-1}}⊕\left(s\right)⊕0_{s^{t-l}},\left(\begin{array}{c} V_{0\cdots 00}\\ V_{0\cdots 01}\\ \vdots \\ V_{\left(s-1)\cdots (s-1)(s-1\right)} \end{array}\right)\right)$$

$$=\left(\begin{array}{cc} \left(0\cdots 00\right)⊕0_{s^{t-l}} & V_{0\cdots 00}\\ \left(0\cdots 01\right)⊕0_{s^{t-l}} & V_{0\cdots 01}\\ \vdots & \vdots \\ \left(\left(s-1\right)\cdots \left(s-1\right)\left(s-1\right)\right)⊕0_{s^{t-l}} & V_{\left(s-1)\cdots (s-1)(s-1\right)} \end{array}\right).\;\;\;\;\;\;\;\;\;\;\;\;\;\;\;\;\;\;\;\;\;\;\;$$

Clearly, *V* is an $OA(s^{t},N,s,t)$ and by Lemma 2, $V_{i_{1}\cdots i_{l-1}i_{l}}$ is also an $OA(s^{t-l},N,s,t-l)$ for $(i_{1},\cdots i_{l-1},i_{l})\in$$Z_{s}^{l}$. Hence, $\left\{V_{0\cdots 00},V_{0\cdots 01},\ldots ,V_{\left(s-1)\cdots (s-1)(s-1\right)}\right\}$ is an orthogonal partition of strength $t^{\prime }$ of *V* where $t^{\prime }=t-l$. By Lemma 5, MD(L)=N+l−t+1. Notice that $MD\left(V_{i_{1}\cdots i_{l-1}i_{l}}\right)=MD\left(L\right)=N+l-t+1\geq t+1\geq t-l+1$. Take h=MD(V)=MD(L)−l=N+1−t≥t−l+1. Then, $d=min\{t^{\prime },h-1\}=t-l$. By Theorem 1, there exists a ${\left(\left(N,s^{l},t-l+1\right)\right)}_{s}$ QECC.

If N+l=2t, then l=N−2(t−l+1−1). So the QECC is optimal. The converse is also true. □

Several infinite families of optimal QECCs can be obtained from Theorem 2.

**Corollary 1.**  *Let s be a prime power. Then there exist optimal QECCs ${\left(\left(3,s,2\right)\right)}_{s}$ for s≥3, ${\left(\left(4,s^{2},2\right)\right)}_{s}$ for s≥5, ${\left(\left(5,s,3\right)\right)}_{s}$ for s≥5, ${\left(\left(6,s^{2},3\right)\right)}_{s}$ for s≥7, ${\left(\left(7,s^{3},3\right)\right)}_{s}$ for s≥9, ${\left(\left(8,s^{2},4\right)\right)}_{s}$ for s≥9, ${\left(\left(9,s^{3},4\right)\right)}_{s}$ for s≥11, ${\left(\left(9,s,5\right)\right)}_{s}$ for s≥9, ${\left(\left(10,s^{2},5\right)\right)}_{s}$ for s≥11, ${\left(\left(11,s,6\right)\right)}_{s}$ for s≥11, and ${\left(\left(12,s^{2},6\right)\right)}_{s}$ for s≥13.*

**Proof.**  In Theorem 2, take t=2, N=3, l=1; t=3, N=4, l=2; t=3, N=5, l=1; t=4, N=6, l=2; t=5, N=7, l=3; t=5, N=8, l=2; t=6, N=9, l=3; t=5, N=9, l=1; t=6, N=10, l=2; t=6, N=11, l=1 and t=7, N=12, l=2, respectively. The desired result follows. □

**Remark 2.**  *Hu et al. have found a suboptimal code ${\left(\left(3,p-1,2\right)\right)}_{p}$ with even p in Ref. [23]. However, from Corollary 1, we can construct optimal QECCs ${\left(\left(3,p,2\right)\right)}_{p}$ such as ${\left(\left(3,4,2\right)\right)}_{4}$ and ${\left(\left(3,8,2\right)\right)}_{8}$, whose basis states are $|\phi_{1}\rangle ,\ldots ,|\phi_{4}\rangle$ and $|\psi_{1}\rangle ,\ldots ,|\psi_{8}\rangle$, respectively, where $|\phi_{1}\rangle \;=|000\rangle +|123\rangle +|231\rangle +|312\rangle$, $|\phi_{2}\rangle \;=|111\rangle +|032\rangle +|320\rangle +|203\rangle$, $|\phi_{3}\rangle \;=|222\rangle +|301\rangle +|013\rangle +|130\rangle$, $|\phi_{4}\rangle \;=|333\rangle +|210\rangle +|102\rangle +|021\rangle$ and $|\psi_{1}\rangle \;=|000\rangle +|123\rangle +|246\rangle +|365\rangle +|451\rangle +|572\rangle +|617\rangle +|734\rangle$, $|\psi_{2}\rangle \;=|111\rangle +|032\rangle +|357\rangle +|274\rangle +|540\rangle +|463\rangle +|706\rangle +|625\rangle$, $|\psi_{3}\rangle \;=|222\rangle +|301\rangle +|064\rangle +|147\rangle +|673\rangle +|750\rangle +|435\rangle +|516\rangle$, $|\psi_{4}\rangle \;=|333\rangle +|210\rangle +|175\rangle +|056\rangle +|762\rangle +|641\rangle +|524\rangle +|407\rangle$, $|\psi_{5}\rangle \;=|444\rangle +|567\rangle +|602\rangle +|721\rangle +|015\rangle +|136\rangle +|253\rangle +|370\rangle$, $|\psi_{6}\rangle \;=|555\rangle +|476\rangle +|713\rangle +|630\rangle +|104\rangle +|027\rangle +|342\rangle +|261\rangle$, $|\psi_{7}\rangle \;=|666\rangle +|745\rangle +|420\rangle +|503\rangle +|237\rangle +|314\rangle +|071\rangle +|152\rangle$, $|\psi_{8}\rangle \;=|777\rangle +|654\rangle +|531\rangle +|412\rangle +|326\rangle +|205\rangle +|160\rangle +|043\rangle$.*

**Theorem 3.**  *(1) If s≥2 is a prime power and s≥t−1≥0, then there exists an ${\left(\left(s+1,s^{t},1\right)\right)}_{s}$ QECC and an ${\left(\left(s+1,1,d+1\right)\right)}_{s}$ QECC where d=min{t,s−t+1}. For any positive integer t and prime power s, a ${\left(\left(t,s^{t},1\right)\right)}_{s}$ QECC can be obtained.*

*(2) If $s=2^{m}$ and m>1, then there exist QECCs ${\left(\left(s+2,1,4\right)\right)}_{s}$, ${\left(\left(s+1,s,3\right)\right)}_{s}$, ${\left(\left(s,s^{2},2\right)\right)}_{s}$ and ${\left(\left(s-1,s^{3},1\right)\right)}_{s}$.*

**Proof.**  (1) If s≥2 is a prime power and s≥t−1≥0, by Lemma 3, an $OA(s^{t},s+1,s,t)$ exists. By Lemma 5, the minimum Hamming distance of this array is s−t+2. Let $K=s^{t}$ in Theorem 1. We have an ${\left(\left(s+1,s^{t},1\right)\right)}_{s}$ QECC. Let K=1 in Theorem 1. We have an ${\left(\left(s+1,1,d+1\right)\right)}_{s}$ QECC where d=min{t,s−t+1}. Similarly, a ${\left(\left(t,s^{t},1\right)\right)}_{s}$ QECC can be obtained since an $OA\left(s^{t},t,s,t\right)=Z_{s}^{t}$ exists.

(2) If $s=2^{m}$, m>1, by Lemma 4, an $OA(s^{3},s+2,s,3)$ exists. By Lemma 5, the minimum Hamming distance of this array is *s*. Let K=1 in Theorem 1. We have an ${\left(\left(s+2,1,4\right)\right)}_{s}$ QECC. Moreover, by Lemma 2, deleting the first column of the $OA(s^{3},s+2,s,3)$, one can have an orthogonal partition $\left\{A_{1},\ldots ,A_{s}\right\}$ of the $OA(s^{3},s+1,s,3)$. By Theorem 1, we have an ${\left(\left(s+1,s,3\right)\right)}_{s}$ QECC. Similarly, deleting the first two or three columns of the $OA(s^{3},s+2,s,3)$, one can have an orthogonal partition $\left\{B_{1},\ldots ,B_{s^{2}}\right\}$ of the $OA(s^{3},s,s,3)$ or an orthogonal partition $\left\{C_{1},\ldots ,C_{s^{3}}\right\}$ of the $OA(s^{3},s-1,s,3)$. By Theorem 1, we have an ${\left(\left(s,s^{2},2\right)\right)}_{s}$ QECC and an ${\left(\left(s-1,s^{3},1\right)\right)}_{s}$ QECC. □

**Remark 3.**  *In Theorem 14 of ref. [12], for 0≤d≤⌊N/2⌋, 3≤N≤s+1 and s>2 (s is a prime power), there exists an ${\left(\left(N,s^{N-2d},d+1\right)\right)}_{s}$ optimal QECC. In this paper, we can obtain not only optimal QECCs with N≤s+1 for any prime power s but also optimal QECCs with N=s+2 for some s. We list all the ${\left(\left(N,K,d+1\right)\right)}_{4}$ and ${\left(\left(N,K,d+1\right)\right)}_{3}$ QECCs constructed in this paper in Table 1, in which the QECCs with N=1,2,6 are not included in ref. [12].*

*In Table 1, (a). $OA\left(4^{2},4,4,2\right)=\left(\left(4\right)⊕0_{4},0_{4}⊕\left(4\right),\left(4\right)⊕\left(4\right),\left(4\right)⊕2\cdot \left(4\right)\right)$. (b). $OA\left(4^{4},5,4,4\right)=\left(0_{4^{3}},0_{4^{2}}⊕\left(4\right),0_{4}⊕\left(4\right)⊕0_{4},\left(4\right)⊕0_{4^{3}},\left(4\right)⊕2\cdot \left(4\right)⊕\left(4\right)\right)⊕\left(4\right)$. (c). $OA\left(3^{3},4,3,3\right)=\left(0_{9},0_{3}⊕\left(3\right),\left(3\right)⊕0_{3},\left(3\right)⊕\left(3\right)\right)⊕\left(3\right)$.*

By using Theorems 1–3 and the propagation rules in ref. [49], we can immediately obtain a general result.

**Corollary 2.**  *For any positive integers N, K, and d satisfying $s^{N-2d}\geq K$, there exists an ${\left(\left(N,K,d+1\right)\right)}_{s}$ QECC for any sufficient large s (power of prime number).*

**Theorem 4.**  *There exist optimal QECCs ${\left(\left(6,5^{2},3\right)\right)}_{5}$ and ${\left(\left(7,7^{3},3\right)\right)}_{7}$.*

**Proof.**  Let s=5 in Lemma 3. $L=OA(5^{4},6,5,4)$ exists. By Lemma 5, MD(L)=3. An orthogonal partition $\left\{L_{1},\ldots ,L_{25}\right\}$ of *L* can be obtained via computer search where each $L_{i}$ is an $OA(5^{2},6,5,2)$. By Theorem 1, there exists a ${\left(\left(6,5^{2},3\right)\right)}_{5}$ QECC.

Similarly, we can obtain a code ${\left(\left(7,7^{3},3\right)\right)}_{7}$. □

We present the explicit construction of the two codes in Appendix A.

In QECC theory, various constructions and propagation rules have been proposed. Based on our QECCs, we can induce an analogous propagation rule for quantum codes.

**Theorem 5.**  *If there exist QECCs ${\left(\left(N,n,d+1\right)\right)}_{s_{1}}$ and ${\left(\left(N,m,d+1\right)\right)}_{s_{2}}$ obtained from Theorems 1–4, then there exists an ${\left(\left(N,nm,d+1\right)\right)}_{s_{1}s_{2}}$ QECC, which can be constructed from Theorem 1.*

**Proof.**  It follows from Lemma 8 and Theorems 1–4. □

**Remark 4.**  *There is a similar result in ref. [16]. However, the theorem above is still constructive.*

The following result is an immediate consequence of Theorem 5.

**Corollary 3.**  *(i) For prime powers $s_{i}\;\left(i=1,\ldots ,n\right)$ and integers N,l,t, if $s=s_{1}\cdots s_{n}$, $2t\leq l+N\leq min\{s_{1},\ldots ,s_{n}\}+1$ and t>l≥1, then there exists an ${\left(\left(N,s^{l},t-l+1\right)\right)}_{s}$ QECC. Moreover, the QECC is optimal if and only if l+N=2t.*

*(ii) Let $s=s_{1}\cdots s_{n}$ for prime powers $s_{1},\ldots ,s_{n}$. Then, there exist optimal QECCs ${\left(\left(3,s,2\right)\right)}_{s}$ for all $s_{i}\geq 3$, ${\left(\left(4,s^{2},2\right)\right)}_{s}$ for all $s_{i}\geq 5$, ${\left(\left(5,s,3\right)\right)}_{s}$ for all $s_{i}\geq 4$, ${\left(\left(6,s^{2},3\right)\right)}_{s}$ for all $s_{i}\geq 5$, ${\left(\left(7,s^{3},3\right)\right)}_{s}$ for all $s_{i}\geq 7$, ${\left(\left(8,s^{2},4\right)\right)}_{s}$ for all $s_{i}\geq 9$, ${\left(\left(9,s^{3},4\right)\right)}_{s}$ for all $s_{i}\geq 11$, ${\left(\left(9,s,5\right)\right)}_{s}$ for all $s_{i}\geq 9$, ${\left(\left(10,s^{2},5\right)\right)}_{s}$ for all $s_{i}\geq 11$, ${\left(\left(11,s,6\right)\right)}_{s}$ for all $s_{i}\geq 11$, and ${\left(\left(12,s^{2},6\right)\right)}_{s}$ for all $s_{i}\geq 13$.*

The comparison of ${\left(\left(6,K,3\right)\right)}_{s}$ and ${\left(\left(7,K,3\right)\right)}_{s}$ QECCs constructed in this paper with the codes in ref. [25] are summarized in Table 2, in which $s_{1},\ldots ,s_{n}$ are all prime powers, O.P. is short for odd prime, and ${\left(\left(7,3,3\right)\right)}_{3}$ and ${\left(\left(7,8,3\right)\right)}_{5}$ are listed in Example 2. In order to distinguish, the codes in ref. [25] are written as ${\left(\left(6,K,3\right)\right)}_{s}^{*}$ and ${\left(\left(7,K,3\right)\right)}_{s}^{*}$ and our codes are denoted by ${\left(\left(6,K,3\right)\right)}_{s}$ and ${\left(\left(7,K,3\right)\right)}_{s}$.

We often use hybrid systems with different dimensions to store, transmit, and process the quantum information. Thus, it is quite necessary to generalize the standard QECCs over a single alphabet to mixed alphabets [24]. The QECCs ${\left(\left(N,K,d\right)\right)}_{s_{1}s_{2}\cdots s_{N}}$ over mixed alphabets have been discussed in refs. [24,39], and the quantum Singleton bound is also generalized. Unfortunately, there are still fewer such studies on quantum codes so far. By the definitions of IrMOA and the *k*-uniform state for heterogeneous systems in refs. [35,36], the method presented here can be generalized to the construction of QECCs over mixed alphabets naturally.

**Theorem 6.**  *For a prime power $s=q^{m}$ and integers l, n, h, t and N with m≥2, 2t≤l+N−n(h−1)≤s+1 and n<N/h, if there exists an OA(s,h,q,t−l) with MD≥1, then there exists an ${\left(\left(N,s^{l},t-l+1\right)\right)}_{s^{N-hn}q^{hn}}$ QECC.*

**Proof.**  Since *s* is a prime power and s≥2t−1, there exists an $OA(s^{t},s+1,s,t)$. Then there exists an $OA(s^{t},l+N-n\left(h-1\right),s,t)$ if l+N−n(h−1)≤s+1.

By Lemma 5, $MD(OA\left(s^{t},l+N-n\left(h-1\right),s,t\right))=l+N-n\left(h-1\right)-t+1$. After permutation of rows, the $OA(s^{t},l+N-n\left(h-1\right),s,t)$ has the following form

$$LL=(\left(s\right)⊕0_{s^{t-1}},0_{s}⊕\left(s\right)⊕0_{s^{t-2}},\ldots ,0_{s^{l-1}}⊕\left(s\right)⊕0_{s^{t-l}},W)$$

$$=\left(\begin{array}{cc} \left(0\cdots 00\right)⊕0_{s^{t-l}} & W_{0\cdots 00}\\ \left(0\cdots 01\right)⊕0_{s^{t-l}} & W_{0\cdots 01}\\ \vdots & \vdots \\ \left(\left(s-1\right)\cdots \left(s-1\right)\left(s-1\right)\right)⊕0_{s^{t-l}} & W_{\left(s-1)\cdots (s-1)(s-1\right)} \end{array}\right).$$

Obviously, *W* is an $OA(s^{t},N-n\left(h-1\right),s,t)$ and by Lemma 2, $W_{i_{1}\cdots i_{l-1}i_{l}}$ is also an $OA(s^{t-l},N-n\left(h-1\right),s,t-l)$ for $\left(i_{1},\cdots i_{l-1},i_{l}\right)\in Z_{s}^{l}$. Hence, $\left\{W_{0\cdots 00},W_{0\cdots 01},\ldots ,W_{\left(s-1)\cdots (s-1)(s-1\right)}\right\}$ is an orthogonal partition of strength $t^{\prime }$ of *W* where $t^{\prime }=t-l$. By Lemma 5, MD(W)=N−n(h−1)−t+1≥t−l+1.

Since there exists an OA(s,h,q,t−l) with MD≥1, by expansive replacement [36], a mixed OA $W^{\prime }=OA\left(s^{t},N,s^{N-hn}q^{nh},t^{\prime }\right)$ can be obtained from the OA *W* by replacing *n*(n<N/h) columns of *s* levels by the following replacement

$$\begin{array}{ccccc} 0 & 1 & \cdots & q^{m}-2 & q^{m}-1\\ ↓ & ↓ & \vdots & ↓ & ↓\\ a_{1} & a_{2} & \cdots & a_{s-1} & a_{s} \end{array}$$

where $a_{1},a_{2},\ldots ,a_{s}$ are all the rows of the $OA(s,h,q,t^{\prime })$. Correspondingly, the strength of $W^{\prime }$ is $t^{\prime }$ and $W^{\prime }$ has an orthogonal partition $\left\{W_{0\cdots 00}^{{}^{\prime }},W_{0\cdots 01}^{{}^{\prime }},\ldots ,W_{\left(s-1)\cdots (s-1)(s-1\right)}^{{}^{\prime }}\right\}$ of strength $t^{\prime }$ where $W_{i_{1}\cdots i_{l-1}i_{l}}^{{}^{\prime }}$ denotes the matrix obtained by replacing the corresponding *n* columns of $W_{i_{1}\cdots i_{l-1}i_{l}}$. Clearly, $MD\left(W^{\prime }\right)\geq N-n\left(h-1\right)-t+1\geq t-l+1$.

By the definition of IrMOA, the $W^{\prime }$ and $W_{i_{1}\cdots i_{l-1}i_{l}}^{{}^{\prime }}$ are IrMOAs of strength t−l, respectively. From the link between IrMOAs and uniform states in ref. [35] and $\left\{W_{0\cdots 00}^{{}^{\prime }},W_{0\cdots 01}^{{}^{\prime }},\ldots ,W_{\left(s-1)\cdots (s-1)(s-1\right)}^{{}^{\prime }}\right\}$, we can obtain $s^{l}$(t−l)-uniform states $|\varphi_{1}\rangle ,\ldots ,|\varphi_{s^{l}}\rangle$, which can be used as an orthogonal basis. Since $min\{t^{\prime },N-n\left(h-1\right)-t\}=t-l$, the complex subspace spanned by the orthogonal basis is an ${\left(\left(N,s^{l},t-l+1\right)\right)}_{s^{N-hn}q^{hn}}$ QECC by a generalization of Theorem 1. □

**Remark 5.**  *If the OA(s,h,q,t−l) in Theorem 6 is replaced by an $OA(s,h,{q_{1}}^{r_{1}}{q_{2}}^{r_{2}}\cdots {q_{v}}^{r_{v}},t-l)$, then the corresponding result is still true.*

## 4. Examples

In this section we shall provide some specific QECCs, which are based on the theorems above.

**Example 1.**  *The optimal code ${\left(\left(5,5,3\right)\right)}_{5}$*

*For the case N=5, t=3, l=1 and s=5, Theorem 2 produces the code ${\left(\left(5,5,3\right)\right)}_{5}$. Here its five basis states are presented.*

*$|\varphi_{1}\rangle \;=00000\rangle +12340\rangle +24130\rangle +31420\rangle +43210\rangle +14411\rangle +21201\rangle +33041\rangle +40331\rangle +02121\rangle +23322\rangle +30112\rangle +42402\rangle +04242\rangle +11032\rangle +32233\rangle +44023\rangle +01313\rangle +13103\rangle +20443\rangle +41144\rangle +03434\rangle +10224\rangle +22014\rangle +34304\rangle$,*

*$|\varphi_{2}\rangle \;=|11110\rangle +|23400\rangle +|30240\rangle +|42030\rangle +|04320\rangle +|20021\rangle +|32311\rangle +|44101\rangle +|01441\rangle +|13231\rangle +|34432\rangle +|41222\rangle +|03012\rangle +|10302\rangle +|22142\rangle +|43343\rangle +|00133\rangle +|12423\rangle +|24213\rangle +|31003\rangle +|02204\rangle +|14044\rangle +|21334\rangle +|33124\rangle +|40414\rangle$,*

*$|\varphi_{3}\rangle \;=|22220\rangle +|34010\rangle +|41300\rangle +|03140\rangle +|10430\rangle +|31131\rangle +|43421\rangle +|00211\rangle +|12001\rangle +|24341\rangle +|40042\rangle +|02332\rangle +|14122\rangle +|21412\rangle +|33202\rangle +|04403\rangle +|11243\rangle +|23033\rangle +|30323\rangle +|42113\rangle +|13314\rangle +|20104\rangle +|32444\rangle +|44234\rangle +|01024\rangle$,*

*$|\varphi_{4}\rangle \;=|33330\rangle +|40120\rangle +|02410\rangle +|14200\rangle +|21040\rangle +|42241\rangle +|04031\rangle +|11321\rangle +|23111\rangle +|30401\rangle +|01102\rangle +|13442\rangle +|20232\rangle +|32022\rangle +|44312\rangle +|10013\rangle +|22303\rangle +|34143\rangle +|41433\rangle +|03223\rangle +|24424\rangle +|31214\rangle +|43004\rangle +|00344\rangle +|12134\rangle$,*

*$|\varphi_{5}\rangle \;=|44440\rangle +|01230\rangle +|13020\rangle +|20310\rangle +|32100\rangle +|03301\rangle +|10141\rangle +|22431\rangle +|34221\rangle +|41011\rangle +|12212\rangle +|24002\rangle +|31342\rangle +|43132\rangle +|00422\rangle +|21123\rangle +|33413\rangle +|40203\rangle +|02043\rangle +|14333\rangle +|30034\rangle +|42324\rangle +|04114\rangle +|11404\rangle +|23244\rangle$.*

**Example 2.**  *Construction of the ${\left(\left(7,3,3\right)\right)}_{3}$ and ${\left(\left(7,8,3\right)\right)}_{5}$ QECCs*

*By Theorem 1, we can obtain two orthogonal bases $\{|\psi_{1}\rangle ,|\psi_{2}\rangle ,|\psi_{3}\rangle \}$ and $\{|\phi_{1}\rangle ,\ldots ,|\phi_{8}\rangle \}$ for ${\left(\left(7,3,3\right)\right)}_{3}$ and ${\left(\left(7,8,3\right)\right)}_{5}$, respectively.*

*$|\psi_{1}\rangle \;=|0000000\rangle +|0001111\rangle +|0110022\rangle +|0112211\rangle +|0221122\rangle +|0222200\rangle +|1011202\rangle +|1012120\rangle +|1120101\rangle +|1121010\rangle +|1200212\rangle +|1202021\rangle +|2020221\rangle +|2022012\rangle +|2101220\rangle +|2102102\rangle +|2210110\rangle +|2211001\rangle$,*

*$|\psi_{2}\rangle \;=|1110000\rangle +|1111111\rangle +|1220022\rangle +|1222211\rangle +|1001122\rangle +|1002200\rangle +|2121202\rangle +|2122120\rangle +|2200101\rangle +|2201010\rangle +|2010212\rangle +|2012021\rangle +|0100221\rangle +|0102012\rangle +|0211220\rangle +|0212102\rangle +|0020110\rangle +|0021001\rangle$,*

*$|\psi_{3}\rangle \;=|2220000\rangle +|2221111\rangle +|2000022\rangle +|2002211\rangle +|2111122\rangle +|2112200\rangle +|0201202\rangle +|0202120\rangle +|0010101\rangle +|0011010\rangle +|0120212\rangle +|0122021\rangle +|1210221\rangle +|1212012\rangle +|1021220\rangle +|1022102\rangle +|1100110\rangle +|1101001\rangle$.*

*$|\phi_{1}\rangle \;=|0001114\rangle +|1112220\rangle +|2223331\rangle +|3334442\rangle +|4440003\rangle +|0001003\rangle +|1112114\rangle +|2223220\rangle +|3334331\rangle +|4440442\rangle +|0001442\rangle +|1112003\rangle +|2223114\rangle +|3334220\rangle +|4440331\rangle +|0001331\rangle +|1112442\rangle +|2223003\rangle +|3334114\rangle +|4440220\rangle +|0001220\rangle +|1112331\rangle +|2223442\rangle +|3334003\rangle +|4440114\rangle +|0124343\rangle +|1230404\rangle +|2341010\rangle +|3402121\rangle +|4013232\rangle +|0124232\rangle +|1230343\rangle +|2341404\rangle +|3402010\rangle +|4013121\rangle +|0124121\rangle +|1230232\rangle +|2341343\rangle +|3402404\rangle +|4013010\rangle +|0124010\rangle +|1230121\rangle +|2341232\rangle +|3402343\rangle +|4013404\rangle +|0124404\rangle +|1230010\rangle +|2341121\rangle +|3402232\rangle +|4013343\rangle +|0242022\rangle +|1303133\rangle +|2414244\rangle +|3020300\rangle +|4131411\rangle +|0242411\rangle +|1303022\rangle +|2414133\rangle +|3020244\rangle +|4131300\rangle +|0242300\rangle +|1303411\rangle +|2414022\rangle +|3020133\rangle +|4131244\rangle +|0242244\rangle +|1303300\rangle +|2414411\rangle +|3020022\rangle +|4131133\rangle +|0242133\rangle +|1303244\rangle +|2414300\rangle +|3020411\rangle +|4131022\rangle +|0310201\rangle +|1421312\rangle +|2032423\rangle +|3143034\rangle +|4204140\rangle +|0310140\rangle +|1421201\rangle +|2032312\rangle +|3143423\rangle +|4204034\rangle +|0310034\rangle +|1421140\rangle +|2032201\rangle +|3143312\rangle +|4204423\rangle +|0310423\rangle +|1421034\rangle +|2032140\rangle +|3143201\rangle +|4204312\rangle +|0310312\rangle +|1421423\rangle +|2032034\rangle +|3143140\rangle +|4204201\rangle +|0433430\rangle +|1044041\rangle +|2100102\rangle +|3211213\rangle +|4322324\rangle +|0433324\rangle +|1044430\rangle +|2100041\rangle +|3211102\rangle +|4322213\rangle +|0433213\rangle +|1044324\rangle +|2100430\rangle +|3211041\rangle +|4322102\rangle +|0433102\rangle +|1044213\rangle +|2100324\rangle +|3211430\rangle +|4322041\rangle +|0433041\rangle +|1044102\rangle +|2100213\rangle +|3211324\rangle +|4322430\rangle$,*

*$|\phi_{2}\rangle \;=|0002011\rangle +|1113122\rangle +|2224233\rangle +|3330344\rangle +|4441400\rangle +|0002400\rangle +|1113011\rangle +|2224122\rangle +|3330233\rangle +|4441344\rangle +|0002344\rangle +|1113400\rangle +|2224011\rangle +|3330122\rangle +|4441233\rangle +|0002233\rangle +|1113344\rangle +|2224400\rangle +|3330011\rangle +|4441122\rangle +|0002122\rangle +|1113233\rangle +|2224344\rangle +|3330400\rangle +|4441011\rangle +|0120240\rangle +|1231301\rangle +|2342412\rangle +|3403023\rangle +|4014134\rangle +|0120134\rangle +|1231240\rangle +|2342301\rangle +|3403412\rangle +|4014023\rangle +|0120023\rangle +|1231134\rangle +|2342240\rangle +|3403301\rangle +|4014412\rangle +|0120412\rangle +|1231023\rangle +|2342134\rangle +|3403240\rangle +|4014301\rangle +|0120301\rangle +|1231412\rangle +|2342023\rangle +|3403134\rangle +|4014240\rangle +|0243424\rangle +|1304030\rangle +|2410141\rangle +|3021202\rangle +|4132313\rangle +|0243313\rangle +|1304424\rangle +|2410030\rangle +|3021141\rangle +|4132202\rangle +|0243202\rangle +|1304313\rangle +|2410424\rangle +|3021030\rangle +|4132141\rangle +|0243141\rangle +|1304202\rangle +|2410313\rangle +|3021424\rangle +|4132030\rangle +|0243030\rangle +|1304141\rangle +|2410202\rangle +|3021313\rangle +|4132424\rangle +|0311103\rangle +|1422214\rangle +|2033320\rangle +|3144431\rangle +|4200042\rangle +|0311042\rangle +|1422103\rangle +|2033214\rangle +|3144320\rangle +|4200431\rangle +|0311431\rangle +|1422042\rangle +|2033103\rangle +|3144214\rangle +|4200320\rangle +|0311320\rangle +|1422431\rangle +|2033042\rangle +|3144103\rangle +|4200214\rangle +|0311214\rangle +|1422320\rangle +|2033431\rangle +|3144042\rangle +|4200103\rangle +|0434332\rangle +|1040443\rangle +|2101004\rangle +|3212110\rangle +|4323221\rangle +|0434221\rangle +|1040332\rangle +|2101443\rangle +|3212004\rangle +|4323110\rangle +|0434110\rangle +|1040221\rangle +|2101332\rangle +|3212443\rangle +|4323004\rangle +|0434004\rangle +|1040110\rangle +|2101221\rangle +|3212332\rangle +|4323443\rangle +|0434443\rangle +|1040004\rangle +|2101110\rangle +|3212221\rangle +|4323332\rangle$,*

*$|\phi_{3}\rangle \;=|0004032\rangle +|1110143\rangle +|2221204\rangle +|3332310\rangle +|4443421\rangle +|0004421\rangle +|1110032\rangle +|2221143\rangle +|3332204\rangle +|4443310\rangle +|0004310\rangle +|1110421\rangle +|2221032\rangle +|3332143\rangle +|4443204\rangle +|0004204\rangle +|1110310\rangle +|2221421\rangle +|3332032\rangle +|4443143\rangle +|0004143\rangle +|1110204\rangle +|2221310\rangle +|3332421\rangle +|4443032\rangle +|0122211\rangle +|1233322\rangle +|2344433\rangle +|3400044\rangle +|4011100\rangle +|0122100\rangle +|1233211\rangle +|2344322\rangle +|3400433\rangle +|4011044\rangle +|0122044\rangle +|1233100\rangle +|2344211\rangle +|3400322\rangle +|4011433\rangle +|0122433\rangle +|1233044\rangle +|2344100\rangle +|3400211\rangle +|4011322\rangle +|0122322\rangle +|1233433\rangle +|2344044\rangle +|3400100\rangle +|4011211\rangle +|0240440\rangle +|1301001\rangle +|2412112\rangle +|3023223\rangle +|4134334\rangle +|0240334\rangle +|1301440\rangle +|2412001\rangle +|3023112\rangle +|4134223\rangle +|0240223\rangle +|1301334\rangle +|2412440\rangle +|3023001\rangle +|4134112\rangle +|0240112\rangle +|1301223\rangle +|2412334\rangle +|3023440\rangle +|4134001\rangle +|0240001\rangle +|1301112\rangle +|2412223\rangle +|3023334\rangle +|4134440\rangle +|0313124\rangle +|1424230\rangle +|2030341\rangle +|3141402\rangle +|4202013\rangle +|0313013\rangle +|1424124\rangle +|2030230\rangle +|3141341\rangle +|4202402\rangle +|0313402\rangle +|1424013\rangle +|2030124\rangle +|3141230\rangle +|4202341\rangle +|0313341\rangle +|1424402\rangle +|2030013\rangle +|3141124\rangle +|4202230\rangle +|0313230\rangle +|1424341\rangle +|2030402\rangle +|3141013\rangle +|4202124\rangle +|0431303\rangle +|1042414\rangle +|2103020\rangle +|3214131\rangle +|4320242\rangle +|0431242\rangle +|1042303\rangle +|2103414\rangle +|3214020\rangle +|4320131\rangle +|0431131\rangle +|1042242\rangle +|2103303\rangle +|3214414\rangle +|4320020\rangle +|0431020\rangle +|1042131\rangle +|2103242\rangle +|3214303\rangle +|4320414\rangle +|0431414\rangle +|1042020\rangle +|2103131\rangle +|3214242\rangle +|4320303\rangle$,*

*$|\phi_{4}\rangle \;=|0010021\rangle +|1121132\rangle +|2232243\rangle +|3343304\rangle +|4404410\rangle +|0010410\rangle +|1121021\rangle +|2232132\rangle +|3343243\rangle +|4404304\rangle +|0010304\rangle +|1121410\rangle +|2232021\rangle +|3343132\rangle +|4404243\rangle +|0010243\rangle +|1121304\rangle +|2232410\rangle +|3343021\rangle +|4404132\rangle +|0010132\rangle +|1121243\rangle +|2232304\rangle +|3343410\rangle +|4404021\rangle +|0133200\rangle +|1244311\rangle +|2300422\rangle +|3411033\rangle +|4022144\rangle +|0133144\rangle +|1244200\rangle +|2300311\rangle +|3411422\rangle +|4022033\rangle +|0133033\rangle +|1244144\rangle +|2300200\rangle +|3411311\rangle +|4022422\rangle +|0133422\rangle +|1244033\rangle +|2300144\rangle +|3411200\rangle +|4022311\rangle +|0133311\rangle +|1244422\rangle +|2300033\rangle +|3411144\rangle +|4022200\rangle +|0201434\rangle +|1312040\rangle +|2423101\rangle +|3034212\rangle +|4140323\rangle +|0201323\rangle +|1312434\rangle +|2423040\rangle +|3034101\rangle +|4140212\rangle +|0201212\rangle +|1312323\rangle +|2423434\rangle +|3034040\rangle +|4140101\rangle +|0201101\rangle +|1312212\rangle +|2423323\rangle +|3034434\rangle +|4140040\rangle +|0201040\rangle +|1312101\rangle +|2423212\rangle +|3034323\rangle +|4140434\rangle +|0324113\rangle +|1430224\rangle +|2041330\rangle +|3102441\rangle +|4213002\rangle +|0324002\rangle +|1430113\rangle +|2041224\rangle +|3102330\rangle +|4213441\rangle +|0324441\rangle +|1430002\rangle +|2041113\rangle +|3102224\rangle +|4213330\rangle +|0324330\rangle +|1430441\rangle +|2041002\rangle +|3102113\rangle +|4213224\rangle +|0324224\rangle +|1430330\rangle +|2041441\rangle +|3102002\rangle +|4213113\rangle +|0442342\rangle +|1003403\rangle +|2114014\rangle +|3220120\rangle +|4331231\rangle +|0442231\rangle +|1003342\rangle +|2114403\rangle +|3220014\rangle +|4331120\rangle +|0442120\rangle +|1003231\rangle +|2114342\rangle +|3220403\rangle +|4331014\rangle +|0442014\rangle +|1003120\rangle +|2114231\rangle +|3220342\rangle +|4331403\rangle +|0442403\rangle +|1003014\rangle +|2114120\rangle +|3220231\rangle +|4331342\rangle$,*

*$|\phi_{5}\rangle \;=|0012002\rangle +|1123113\rangle +|2234224\rangle +|3340330\rangle +|4401441\rangle +|0012441\rangle +|1123002\rangle +|2234113\rangle +|3340224\rangle +|4401330\rangle +|0012330\rangle +|1123441\rangle +|2234002\rangle +|3340113\rangle +|4401224\rangle +|0012224\rangle +|1123330\rangle +|2234441\rangle +|3340002\rangle +|4401113\rangle +|0012113\rangle +|1123224\rangle +|2234330\rangle +|3340441\rangle +|4401002\rangle +|0130231\rangle +|1241342\rangle +|2302403\rangle +|3413014\rangle +|4024120\rangle +|0130120\rangle +|1241231\rangle +|2302342\rangle +|3413403\rangle +|4024014\rangle +|0130014\rangle +|1241120\rangle +|2302231\rangle +|3413342\rangle +|4024403\rangle +|0130403\rangle +|1241014\rangle +|2302120\rangle +|3413231\rangle +|4024342\rangle +|0130342\rangle +|1241403\rangle +|2302014\rangle +|3413120\rangle +|4024231\rangle +|0203410\rangle +|1314021\rangle +|2420132\rangle +|3031243\rangle +|4142304\rangle +|0203304\rangle +|1314410\rangle +|2420021\rangle +|3031132\rangle +|4142243\rangle +|0203243\rangle +|1314304\rangle +|2420410\rangle +|3031021\rangle +|4142132\rangle +|0203132\rangle +|1314243\rangle +|2420304\rangle +|3031410\rangle +|4142021\rangle +|0203021\rangle +|1314132\rangle +|2420243\rangle +|3031304\rangle +|4142410\rangle +|0321144\rangle +|1432200\rangle +|2043311\rangle +|3104422\rangle +|4210033\rangle +|0321033\rangle +|1432144\rangle +|2043200\rangle +|3104311\rangle +|4210422\rangle +|0321422\rangle +|1432033\rangle +|2043144\rangle +|3104200\rangle +|4210311\rangle +|0321311\rangle +|1432422\rangle +|2043033\rangle +|3104144\rangle +|4210200\rangle +|0321200\rangle +|1432311\rangle +|2043422\rangle +|3104033\rangle +|4210144\rangle +|0444323\rangle +|1000434\rangle +|2111040\rangle +|3222101\rangle +|4333212\rangle +|0444212\rangle +|1000323\rangle +|2111434\rangle +|3222040\rangle +|4333101\rangle +|0444101\rangle +|1000212\rangle +|2111323\rangle +|3222434\rangle +|4333040\rangle +|0444040\rangle +|1000101\rangle +|2111212\rangle +|3222323\rangle +|4333434\rangle +|0444434\rangle +|1000040\rangle +|2111101\rangle +|3222212\rangle +|4333323\rangle$,*

*$|\phi_{6}\rangle \;=|0021012\rangle +|1132123\rangle +|2243234\rangle +|3304340\rangle +|4410401\rangle +|0021401\rangle +|1132012\rangle +|2243123\rangle +|3304234\rangle +|4410340\rangle +|0021340\rangle +|1132401\rangle +|2243012\rangle +|3304123\rangle +|4410234\rangle +|0021234\rangle +|1132340\rangle +|2243401\rangle +|3304012\rangle +|4410123\rangle +|0021123\rangle +|1132234\rangle +|2243340\rangle +|3304401\rangle +|4410012\rangle +|0144241\rangle +|1200302\rangle +|2311413\rangle +|3422024\rangle +|4033130\rangle +|0144130\rangle +|1200241\rangle +|2311302\rangle +|3422413\rangle +|4033024\rangle +|0144024\rangle +|1200130\rangle +|2311241\rangle +|3422302\rangle +|4033413\rangle +|0144413\rangle +|1200024\rangle +|2311130\rangle +|3422241\rangle +|4033302\rangle +|0144302\rangle +|1200413\rangle +|2311024\rangle +|3422130\rangle +|4033241\rangle +|0212420\rangle +|1323031\rangle +|2434142\rangle +|3040203\rangle +|4101314\rangle +|0212314\rangle +|1323420\rangle +|2434031\rangle +|3040142\rangle +|4101203\rangle +|0212203\rangle +|1323314\rangle +|2434420\rangle +|3040031\rangle +|4101142\rangle +|0212142\rangle +|1323203\rangle +|2434314\rangle +|3040420\rangle +|4101031\rangle +|0212031\rangle +|1323142\rangle +|2434203\rangle +|3040314\rangle +|4101420\rangle +|0330104\rangle +|1441210\rangle +|2002321\rangle +|3113432\rangle +|4224043\rangle +|0330043\rangle +|1441104\rangle +|2002210\rangle +|3113321\rangle +|4224432\rangle +|0330432\rangle +|1441043\rangle +|2002104\rangle +|3113210\rangle +|4224321\rangle +|0330321\rangle +|1441432\rangle +|2002043\rangle +|3113104\rangle +|4224210\rangle +|0330210\rangle +|1441321\rangle +|2002432\rangle +|3113043\rangle +|4224104\rangle +|0403333\rangle +|1014444\rangle +|2120000\rangle +|3231111\rangle +|4342222\rangle +|0403222\rangle +|1014333\rangle +|2120444\rangle +|3231000\rangle +|4342111\rangle +|0403111\rangle +|1014222\rangle +|2120333\rangle +|3231444\rangle +|4342000\rangle +|0403000\rangle +|1014111\rangle +|2120222\rangle +|3231333\rangle +|4342444\rangle +|0403444\rangle +|1014000\rangle +|2120111\rangle +|3231222\rangle +|4342333\rangle$,*

*$|\phi_{7}\rangle \;=|0023020\rangle +|1134131\rangle +|2240242\rangle +|3301303\rangle +|4412414\rangle +|0023414\rangle +|1134020\rangle +|2240131\rangle +|3301242\rangle +|4412303\rangle +|0023303\rangle +|1134414\rangle +|2240020\rangle +|3301131\rangle +|4412242\rangle +|0023242\rangle +|1134303\rangle +|2240414\rangle +|3301020\rangle +|4412131\rangle +|0023131\rangle +|1134242\rangle +|2240303\rangle +|3301414\rangle +|4412020\rangle +|0141204\rangle +|1202310\rangle +|2313421\rangle +|3424032\rangle +|4030143\rangle +|0141143\rangle +|1202204\rangle +|2313310\rangle +|3424421\rangle +|4030032\rangle +|0141032\rangle +|1202143\rangle +|2313204\rangle +|3424310\rangle +|4030421\rangle +|0141421\rangle +|1202032\rangle +|2313143\rangle +|3424204\rangle +|4030310\rangle +|0141310\rangle +|1202421\rangle +|2313032\rangle +|3424143\rangle +|4030204\rangle +|0214433\rangle +|1320044\rangle +|2431100\rangle +|3042211\rangle +|4103322\rangle +|0214322\rangle +|1320433\rangle +|2431044\rangle +|3042100\rangle +|4103211\rangle +|0214211\rangle +|1320322\rangle +|2431433\rangle +|3042044\rangle +|4103100\rangle +|0214100\rangle +|1320211\rangle +|2431322\rangle +|3042433\rangle +|4103044\rangle +|0214044\rangle +|1320100\rangle +|2431211\rangle +|3042322\rangle +|4103433\rangle +|0332112\rangle +|1443223\rangle +|2004334\rangle +|3110440\rangle +|4221001\rangle +|0332001\rangle +|1443112\rangle +|2004223\rangle +|3110334\rangle +|4221440\rangle +|0332440\rangle +|1443001\rangle +|2004112\rangle +|3110223\rangle +|4221334\rangle +|0332334\rangle +|1443440\rangle +|2004001\rangle +|3110112\rangle +|4221223\rangle +|0332223\rangle +|1443334\rangle +|2004440\rangle +|3110001\rangle +|4221112\rangle +|0400341\rangle +|1011402\rangle +|2122013\rangle +|3233124\rangle +|4344230\rangle +|0400230\rangle +|1011341\rangle +|2122402\rangle +|3233013\rangle +|4344124\rangle +|0400124\rangle +|1011230\rangle +|2122341\rangle +|3233402\rangle +|4344013\rangle +|0400013\rangle +|1011124\rangle +|2122230\rangle +|3233341\rangle +|4344402\rangle +|0400402\rangle +|1011013\rangle +|2122124\rangle +|3233230\rangle +|4344341\rangle$,*

*$|\phi_{8}\rangle \;=|0030444\rangle +|1141000\rangle +|2202111\rangle +|3313222\rangle +|4424333\rangle +|0030333\rangle +|1141444\rangle +|2202000\rangle +|3313111\rangle +|4424222\rangle +|0030222\rangle +|1141333\rangle +|2202444\rangle +|3313000\rangle +|4424111\rangle +|0030111\rangle +|1141222\rangle +|2202333\rangle +|3313444\rangle +|4424000\rangle +|0030000\rangle +|1141111\rangle +|2202222\rangle +|3313333\rangle +|4424444\rangle +|0103123\rangle +|1214234\rangle +|2320340\rangle +|3431401\rangle +|4042012\rangle +|0103012\rangle +|1214123\rangle +|2320234\rangle +|3431340\rangle +|4042401\rangle +|0103401\rangle +|1214012\rangle +|2320123\rangle +|3431234\rangle +|4042340\rangle +|0103340\rangle +|1214401\rangle +|2320012\rangle +|3431123\rangle +|4042234\rangle +|0103234\rangle +|1214340\rangle +|2320401\rangle +|3431012\rangle +|4042123\rangle +|0221302\rangle +|1332413\rangle +|2443024\rangle +|3004130\rangle +|4110241\rangle +|0221241\rangle +|1332302\rangle +|2443413\rangle +|3004024\rangle +|4110130\rangle +|0221130\rangle +|1332241\rangle +|2443302\rangle +|3004413\rangle +|4110024\rangle +|0221024\rangle +|1332130\rangle +|2443241\rangle +|3004302\rangle +|4110413\rangle +|0221413\rangle +|1332024\rangle +|2443130\rangle +|3004241\rangle +|4110302\rangle +|0344031\rangle +|1400142\rangle +|2011203\rangle +|3122314\rangle +|4233420\rangle +|0344420\rangle +|1400031\rangle +|2011142\rangle +|3122203\rangle +|4233314\rangle +|0344314\rangle +|1400420\rangle +|2011031\rangle +|3122142\rangle +|4233203\rangle +|0344203\rangle +|1400314\rangle +|2011420\rangle +|3122031\rangle +|4233142\rangle +|0344142\rangle +|1400203\rangle +|2011314\rangle +|3122420\rangle +|4233031\rangle +|0412210\rangle +|1023321\rangle +|2134432\rangle +|3240043\rangle +|4301104\rangle +|0412104\rangle +|1023210\rangle +|2134321\rangle +|3240432\rangle +|4301043\rangle +|0412043\rangle +|1023104\rangle +|2134210\rangle +|3240321\rangle +|4301432\rangle +|0412432\rangle +|1023043\rangle +|2134104\rangle +|3240210\rangle +|4301321\rangle +|0412321\rangle +|1023432\rangle +|2134043\rangle +|3240104\rangle +|4301210\rangle$.*

**Example 3.**  *Construction of QECCs ${\left(\left(8,9,3\right)\right)}_{9^{4}3^{4}}$, ${\left(\left(7,9,3\right)\right)}_{9^{4}3^{3}}$, ${\left(\left(6,9,3\right)\right)}_{9^{4}3^{2}}$, ${\left(\left(4+n_{1},16,3\right)\right)}_{16^{4}4^{n_{1}}}$ for $2\leq n_{1}\leq 5$, ${\left(\left(5+n_{2},16,3\right)\right)}_{16^{4}4^{1}2^{n_{2}}}$ for $2\leq n_{2}\leq 12$, ${\left(\left(6+n_{2},16,3\right)\right)}_{16^{4}4^{2}2^{n_{2}}}$ for $0\leq n_{2}\leq 9$, ${\left(\left(7+n_{2},16,3\right)\right)}_{16^{4}4^{3}2^{n_{2}}}$ for $0\leq n_{2}\leq 6$, ${\left(\left(8+n_{2},16,3\right)\right)}_{16^{4}4^{4}2^{n_{2}}}$ for $0\leq n_{2}\leq 3$, ${\left(\left(4+n_{2},16,3\right)\right)}_{16^{4}2^{n_{2}}}$ for $4\leq n_{2}\leq 15$, ${\left(\left(5+n_{2},16,3\right)\right)}_{16^{4}8^{1}2^{n_{2}}}$ for $1\leq n_{2}\leq 8$.*

*(1) Consider the codes ${\left(\left(8,9,3\right)\right)}_{9^{4}3^{4}}$, ${\left(\left(7,9,3\right)\right)}_{9^{4}3^{3}}$ and the optimal code ${\left(\left(6,9,3\right)\right)}_{9^{4}3^{2}}$.*

*Take q=3, m=2, l=1, n=1, t=3, h=4 and N=8 in Theorem 6. Since there exists an $OA\left(s,h,q,t-l\right)=OA\left(9,4,3,2\right)={\left(\begin{array}{ccccccccc} 0 & 0 & 0 & 1 & 1 & 1 & 2 & 2 & 2\\ 0 & 1 & 2 & 0 & 1 & 2 & 0 & 1 & 2\\ 0 & 1 & 2 & 1 & 2 & 0 & 2 & 0 & 1\\ 0 & 1 & 2 & 2 & 0 & 1 & 1 & 2 & 0 \end{array}\right)}^{T}$, we have the replacement:*

$$\begin{array}{ccccc} 0 & 1 & \cdots & 7 & 8\\ ↓ & ↓ & \vdots & ↓ & ↓\\ 0000 & 0111 & \cdots & 2102 & 2210 \end{array}.$$

*Then, we obtain the code ${\left(\left(8,9,3\right)\right)}_{9^{4}3^{4}}$.*

*Obviously, there exist the arrays OA(9,3,3,2) and OA(9,2,3,2). Similarly, we can have the codes ${\left(\left(7,9,3\right)\right)}_{9^{4}3^{3}}$ and ${\left(\left(6,9,3\right)\right)}_{9^{4}3^{2}}$. According to the quantum Singleton bound of QECC over mixed alphabets [39], the code ${\left(\left(6,9,3\right)\right)}_{9^{4}3^{2}}$ is optimal.*

*(2) Consider the construction of the remaining codes.*

*Take q=2, m=4, l=1, n=1, t=3 in Theorem 6. Using $OA(16,n_{1},4,2)$ for $2\leq n_{1}\leq 5$, $OA(16,4^{1}2^{n_{2}},2)$ for $2\leq n_{2}\leq 12$, $OA(16,4^{2}2^{n_{2}},2)$ for $0\leq n_{2}\leq 9$, $OA(16,4^{3}2^{n_{2}},2)$ for $0\leq n_{2}\leq 6$, $OA(16,4^{4}2^{n_{2}},2)$ for $0\leq n_{2}\leq 3$, $OA(16,n_{2},2,2)$ for $4\leq n_{2}\leq 15$, $OA(16,8^{1}2^{n_{2}},2)$ for $1\leq n_{2}\leq 8$ as the OA(s,h,q,t−l) in Theorem 6, respectively, we can obtain the desired codes. According to the quantum Singleton bound of QECC over mixed alphabets [39], the codes ${\left(\left(6,16,3\right)\right)}_{16^{4}4^{2}}$, ${\left(\left(7,16,3\right)\right)}_{16^{4}4^{1}2^{2}}$, ${\left(\left(8,16,3\right)\right)}_{16^{4}2^{4}}$ and ${\left(\left(6,16,3\right)\right)}_{16^{4}8^{1}2^{1}}$ are optimal.*

**Remark 6.**  *Given the optimal ${\left(\left(3,K,2\right)\right)}_{4^{n_{1}}2^{n_{2}}}$ codes and the trivial codes ${\left(\left(2,1,2\right)\right)}_{4}$ and ${\left(\left(2,1,2\right)\right)}_{2}$, Wang et al. constructed most of the optimal ${\left(\left(N,K,2\right)\right)}_{4^{n_{1}}2^{n_{2}}}$ codes via stabilizer pasting [24]. Obviously, the parameters d, s and q of QECCs ${\left(\left(N,K,d+1\right)\right)}_{s^{n_{1}}q^{n_{2}}}$ obtained by Theorem 6 are more flexible than that in ref. [24].*

## 5. Conclusions

This paper studied the relation between QECCs and OAs and presented a general method of constructing QECCs. Compared to previous constructions, our technique has some interesting features.

(1) The results are not just existence results, but constructive results. A lot of families of QECCs over a single alphabet and over mixed alphabets, including families of optimal codes, can explicitly be obtained, and are not limited to the classes listed in the paper.

(2) All the constructed QECCs are pure.

(3) Each basis state of these codes has far less terms.

(4) Some optimal QECCs ${\left(\left(N,K,2\right)\right)}_{s}$ for an odd *N* with *s* can even be constructed, such as ${\left(\left(3,4,2\right)\right)}_{4}$, ${\left(\left(3,8,2\right)\right)}_{8}$ and ${\left(\left(5,8^{3},2\right)\right)}_{8}$ compared with the codes in [23].

(5) For any positive integers *N*, *K* and *d* satisfying $s^{N-2d}\geq K$, there exist QECCs ${\left(\left(N,K,d+1\right)\right)}_{s}$, naturally including optimal codes, for any sufficient large *s*, not necessarily equal to a prime power.

(6) A quantum code constructed in this paper can easily produce uniform states.

The theory of quantum information often benefits from OAs. Next we will study how to use OAs with special properties to construct new QECCs. Notice that the knowledge on QECCs over mixed alphabets remains rather limited so far. Therefore, how to use mixed OAs to construct such QECCs will also be our work in the future. All QECCs in our paper are explicitly given, which can provide great convenience for users. By means of their stabilizer matrices, the QECCs can be used to correct errors such as existing quantum codes. Furthermore, we will try to explore a new and simple way to correct errors in the future.

## Figures and Tables

**Table 1:** ${\left(\left(N,K,d+1\right)\right)}_{4}$ and ${\left(\left(N,K,d+1\right)\right)}_{3}$ QECCs constructed in this paper.

${\left(\left(N,K,d+1\right)\right)}_{4}$ QECC	Reference	Parameters	${\left(\left(N,K,d+1\right)\right)}_{3}$ QECC	Reference	Parameters
${\left(\left(1,4,1\right)\right)}_{4}$	Theorem 2	l=1, t=1	${\left(\left(1,3,1\right)\right)}_{3}$	Theorem 2	l=1, t=1
${\left(\left(2,4^{2},1\right)\right)}_{4}$	Theorem 2	l=2, t=2	${\left(\left(2,3^{2},1\right)\right)}_{3}$	Theorem 2	l=2, t=2
${\left(\left(3,4^{3},1\right)\right)}_{4}$	Theorem 3 (2)	m=2	${\left(\left(3,3,2\right)\right)}_{3}$	Corollary 1	
${\left(\left(3,4^{1},2\right)\right)}_{4}$	Corollary 1		${\left(\left(3,3^{3},1\right)\right)}_{3}$	Theorem 3 (1)	t=3
${\left(\left(4,4^{4},1\right)\right)}_{4}$	Theorem 3 (1)	t=4	${\left(\left(4,3^{4},1\right)\right)}_{3}$	Theorem 3 (1)	t=3
${\left(\left(4,4^{2},2\right)\right)}_{4}$	Theorem 3 (2)	m=2	${\left(\left(4,3^{2},2\right)\right)}_{3}$	Theorem 1	$OA{\left(3^{3},4,3,3\right)}^{\left(c\right)}$,
${\left(\left(4,1,3\right)\right)}_{4}$	Theorem 1	$OA{\left(4^{2},4,4,2\right)}^{\left(a\right)}$,			h=2
		h=3	${\left(\left(4,1,3\right)\right)}_{3}$	Theorem 3 (1)	t=2
${\left(\left(5,4^{5},1\right)\right)}_{4}$	Theorem 3 (1)	t=5			
${\left(\left(5,4^{3},2\right)\right)}_{4}$	Theorem 1	$OA{\left(4^{4},5,4,4\right)}^{\left(b\right)}$,	${\left(\left(5,4,3\right)\right)}_{4}$	Theorem 3 (2)	m=2
		h=2	${\left(\left(6,1,4\right)\right)}_{4}$	Theorem 3 (2)	m=2

**Table 2:** Comparison of the constructed ${\left(\left(6,K,3\right)\right)}_{s}$ and ${\left(\left(7,K,3\right)\right)}_{s}$ QECCs with such codes in ref. [25].

	*s* and *K*	*s* and *K* of	The Number of Terms
	of Optimal QECC	Unoptimal QECC	in a Basis State
${\left(\left(6,K,3\right)\right)}_{s}^{*}$	O.P. s≥3, $K=s^{2}$		$s^{6}$
${\left(\left(7,K,3\right)\right)}_{s}^{*}$	O.P. s≥3, $K=s^{3}$		$s^{7}$
${\left(\left(6,K,3\right)\right)}_{s}$	$s=s_{1}\cdots s_{n}$, all $s_{i}\geq 5$,$K=s^{2}$		$s^{2}$
		s=3, K=1	$2s^{2}$
${\left(\left(7,K,3\right)\right)}_{s}$	$s=s_{1}\cdots s_{n}$, all $s_{i}\geq 7$, $K=s^{3}$		$s^{2}$
		s=3, K=3	$s^{2}$
		s=5, K=8	$s^{2}$

## Data Availability

Not applicable.

## Appendix A

(I) The optimal code ${\left(\left(6,5^{2},3\right)\right)}_{5}$ in Theorem 4.

We list its 25 basis states as follows.

$|\varphi_{1}\rangle =|000000\rangle +|011111\rangle +|022222\rangle +|033333\rangle +|044444\rangle +|101234\rangle +|112340\rangle +|123401\rangle +|134012\rangle +|140123\rangle +|202413\rangle +|213024\rangle +|224130\rangle +|230241\rangle +|241302\rangle +|303142\rangle +|314203\rangle +|320314\rangle +|331420\rangle +|342031\rangle +|404321\rangle +|410432\rangle +|421043\rangle +|432104\rangle +|443210\rangle$,

$|\varphi_{2}\rangle =|001324\rangle +|012430\rangle +|023041\rangle +|034102\rangle +|040213\rangle +|102003\rangle +|113114\rangle +|124220\rangle +|130331\rangle +|141442\rangle +|203232\rangle +|214343\rangle +|220404\rangle +|231010\rangle +|242121\rangle +|304411\rangle +|310022\rangle +|321133\rangle +|332244\rangle +|343300\rangle +|400140\rangle +|411201\rangle +|422312\rangle +|433423\rangle +|444034\rangle$,

$|\varphi_{3}\rangle =|002143\rangle +|013204\rangle +|024310\rangle +|030421\rangle +|041032\rangle +|103322\rangle +|114433\rangle +|120044\rangle +|131100\rangle +|142211\rangle +|204001\rangle +|210112\rangle +|221223\rangle +|232334\rangle +|243440\rangle +|300230\rangle +|311341\rangle +|322402\rangle +|333013\rangle +|344124\rangle +|401414\rangle +|412020\rangle +|423131\rangle +|434242\rangle +|440303\rangle$,

$|\varphi_{4}\rangle =|003412\rangle +|014023\rangle +|020134\rangle +|031240\rangle +|042301\rangle +|104141\rangle +|110202\rangle +|121313\rangle +|132424\rangle +|143030\rangle +|200320\rangle +|211431\rangle +|222042\rangle +|233103\rangle +|244214\rangle +|301004\rangle +|312110\rangle +|323221\rangle +|334332\rangle +|340443\rangle +|402233\rangle +|413344\rangle +|424400\rangle +|430011\rangle +|441122\rangle$,

$|\varphi_{5}\rangle =|004231\rangle +|010342\rangle +|021403\rangle +|032014\rangle +|043120\rangle +|100410\rangle +|111021\rangle +|122132\rangle +|133243\rangle +|144304\rangle +|201144\rangle +|212200\rangle +|223311\rangle +|234422\rangle +|240033\rangle +|302323\rangle +|313434\rangle +|324040\rangle +|330101\rangle +|341212\rangle +|403002\rangle +|414113\rangle +|420224\rangle +|431330\rangle +|442441\rangle$,

$|\varphi_{6}\rangle =|001441\rangle +|012002\rangle +|023113\rangle +|034224\rangle +|040330\rangle +|102120\rangle +|113231\rangle +|124342\rangle +|130403\rangle +|141014\rangle +|203304\rangle +|214410\rangle +|220021\rangle +|231132\rangle +|242243\rangle +|304033\rangle +|310144\rangle +|321200\rangle +|332311\rangle +|343422\rangle +|400212\rangle +|411323\rangle +|422434\rangle +|433040\rangle +|444101\rangle$,

$|\varphi_{7}\rangle =|002210\rangle +|013321\rangle +|024432\rangle +|030043\rangle +|041104\rangle +|103444\rangle +|114000\rangle +|120111\rangle +|131222\rangle +|142333\rangle +|204123\rangle +|210234\rangle +|221340\rangle +|232401\rangle +|243012\rangle +|300302\rangle +|311413\rangle +|322024\rangle +|333130\rangle +|344241\rangle +|401031\rangle +|412142\rangle +|423203\rangle +|434314\rangle +|440420\rangle$,

$|\varphi_{8}\rangle =|003034\rangle +|014140\rangle +|020201\rangle +|031312\rangle +|042423\rangle +|104213\rangle +|110324\rangle +|121430\rangle +|132041\rangle +|143102\rangle +|200442\rangle +|211003\rangle +|222114\rangle +|233220\rangle +|244331\rangle +|301121\rangle +|312232\rangle +|323343\rangle +|334404\rangle +|340010\rangle +|402300\rangle +|413411\rangle +|424022\rangle +|430133\rangle +|441244\rangle$,

$|\varphi_{9}\rangle =|004303\rangle +|010414\rangle +|021020\rangle +|032131\rangle +|043242\rangle +|100032\rangle +|111143\rangle +|122204\rangle +|133310\rangle +|144421\rangle +|201211\rangle +|212322\rangle +|223433\rangle +|234044\rangle +|240100\rangle +|302440\rangle +|313001\rangle +|324112\rangle +|330223\rangle +|341334\rangle +|403124\rangle +|414230\rangle +|420341\rangle +|431402\rangle +|442013\rangle$,

$|\varphi_{10}\rangle =|000122\rangle +|011233\rangle +|022344\rangle +|033400\rangle +|044011\rangle +|101301\rangle +|112412\rangle +|123023\rangle +|134134\rangle +|140240\rangle +|202030\rangle +|213141\rangle +|224202\rangle +|230313\rangle +|241424\rangle +|303214\rangle +|314320\rangle +|320431\rangle +|331042\rangle +|342103\rangle +|404443\rangle +|410004\rangle +|421110\rangle +|432221\rangle +|443332\rangle$,

$|\varphi_{11}\rangle =|002332\rangle +|013443\rangle +|024004\rangle +|030110\rangle +|041221\rangle +|103011\rangle +|114122\rangle +|120233\rangle +|131344\rangle +|142400\rangle +|204240\rangle +|210301\rangle +|221412\rangle +|232023\rangle +|243134\rangle +|300424\rangle +|311030\rangle +|322141\rangle +|333202\rangle +|344313\rangle +|401103\rangle +|412214\rangle +|423320\rangle +|434431\rangle +|440042\rangle$,

$|\varphi_{12}\rangle =|003101\rangle +|014212\rangle +|020323\rangle +|031434\rangle +|042040\rangle +|104330\rangle +|110441\rangle +|121002\rangle +|132113\rangle +|143224\rangle +|200014\rangle +|211120\rangle +|222231\rangle +|233342\rangle +|244403\rangle +|301243\rangle +|312304\rangle +|323410\rangle +|334021\rangle +|340132\rangle +|402422\rangle +|413033\rangle +|424144\rangle +|430200\rangle +|441311\rangle$,

$|\varphi_{13}\rangle =|004420\rangle +|010031\rangle +|021142\rangle +|032203\rangle +|043314\rangle +|100104\rangle +|111210\rangle +|122321\rangle +|133432\rangle +|144043\rangle +|201333\rangle +|212444\rangle +|223000\rangle +|234111\rangle +|240222\rangle +|302012\rangle +|313123\rangle +|324234\rangle +|330340\rangle +|341401\rangle +|403241\rangle +|414302\rangle +|420413\rangle +|431024\rangle +|442130\rangle$,

$|\varphi_{14}\rangle =|000244\rangle +|011300\rangle +|022411\rangle +|033022\rangle +|044133\rangle +|101423\rangle +|112034\rangle +|123140\rangle +|134201\rangle +|140312\rangle +|202102\rangle +|213213\rangle +|224324\rangle +|230430\rangle +|241041\rangle +|303331\rangle +|314442\rangle +|320003\rangle +|331114\rangle +|342220\rangle +|404010\rangle +|410121\rangle +|421232\rangle +|432343\rangle +|443404\rangle$,

$|\varphi_{15}\rangle =|001013\rangle +|012124\rangle +|023230\rangle +|034341\rangle +|040402\rangle +|102242\rangle +|113303\rangle +|124414\rangle +|130020\rangle +|141131\rangle +|203421\rangle +|214032\rangle +|220143\rangle +|231204\rangle +|242310\rangle +|304100\rangle +|310211\rangle +|321322\rangle +|332433\rangle +|343044\rangle +|400334\rangle +|411440\rangle +|422001\rangle +|433112\rangle +|444223\rangle$,

$|\varphi_{16}\rangle =|003223\rangle +|014334\rangle +|020440\rangle +|031001\rangle +|042112\rangle +|104402\rangle +|110013\rangle +|121124\rangle +|132230\rangle +|143341\rangle +|200131\rangle +|211242\rangle +|222303\rangle +|233414\rangle +|244020\rangle +|301310\rangle +|312421\rangle +|323032\rangle +|334143\rangle +|340204\rangle +|402044\rangle +|413100\rangle +|424211\rangle +|430322\rangle +|441433\rangle$,

$|\varphi_{17}\rangle =|004042\rangle +|010103\rangle +|021214\rangle +|032320\rangle +|043431\rangle +|100221\rangle +|111332\rangle +|122443\rangle +|133004\rangle +|144110\rangle +|201400\rangle +|212011\rangle +|223122\rangle +|234233\rangle +|240344\rangle +|302134\rangle +|313240\rangle +|324301\rangle +|330412\rangle +|341023\rangle +|403313\rangle +|414424\rangle +|420030\rangle +|431141\rangle +|442202\rangle$,

$|\varphi_{18}\rangle =|000311\rangle +|011422\rangle +|022033\rangle +|033144\rangle +|044200\rangle +|101040\rangle +|112101\rangle +|123212\rangle +|134323\rangle +|140434\rangle +|202224\rangle +|213330\rangle +|224441\rangle +|230002\rangle +|241113\rangle +|303403\rangle +|314014\rangle +|320120\rangle +|331231\rangle +|342342\rangle +|404132\rangle +|410243\rangle +|421304\rangle +|432410\rangle +|443021\rangle$,

$|\varphi_{19}\rangle =|001130\rangle +|012241\rangle +|023302\rangle +|034413\rangle +|040024\rangle +|102314\rangle +|113420\rangle +|124031\rangle +|130142\rangle +|141203\rangle +|203043\rangle +|214104\rangle +|220210\rangle +|231321\rangle +|242432\rangle +|304222\rangle +|310333\rangle +|321444\rangle +|332000\rangle +|343111\rangle +|400401\rangle +|411012\rangle +|422123\rangle +|433234\rangle +|444340\rangle$,

$|\varphi_{20}\rangle =|002404\rangle +|013010\rangle +|024121\rangle +|030232\rangle +|041343\rangle +|103133\rangle +|114244\rangle +|120300\rangle +|131411\rangle +|142022\rangle +|204312\rangle +|210423\rangle +|221034\rangle +|232140\rangle +|243201\rangle +|300041\rangle +|311102\rangle +|322213\rangle +|333324\rangle +|344430\rangle +|401220\rangle +|412331\rangle +|423442\rangle +|434003\rangle +|440114\rangle$,

$|\varphi_{21}\rangle =|004114\rangle +|010220\rangle +|021331\rangle +|032442\rangle +|043003\rangle +|100343\rangle +|111404\rangle +|122010\rangle +|133121\rangle +|144232\rangle +|201022\rangle +|212133\rangle +|223244\rangle +|234300\rangle +|240411\rangle +|302201\rangle +|313312\rangle +|324423\rangle +|330034\rangle +|341140\rangle +|403430\rangle +|414041\rangle +|420102\rangle +|431213\rangle +|442324\rangle$,

$|\varphi_{22}\rangle =|000433\rangle +|011044\rangle +|022100\rangle +|033211\rangle +|044322\rangle +|101112\rangle +|112223\rangle +|123334\rangle +|134440\rangle +|140001\rangle +|202341\rangle +|213402\rangle +|224013\rangle +|230124\rangle +|241230\rangle +|303020\rangle +|314131\rangle +|320242\rangle +|331303\rangle +|342414\rangle +|404204\rangle +|410310\rangle +|421421\rangle +|432032\rangle +|443143\rangle$,

$|\varphi_{23}\rangle =|001202\rangle +|012313\rangle +|023424\rangle +|034030\rangle +|040141\rangle +|102431\rangle +|113042\rangle +|124103\rangle +|130214\rangle +|141320\rangle +|203110\rangle +|214221\rangle +|220332\rangle +|231443\rangle +|242004\rangle +|304344\rangle +|310400\rangle +|321011\rangle +|332122\rangle +|343233\rangle +|400023\rangle +|411134\rangle +|422240\rangle +|433301\rangle +|444412\rangle$,

$|\varphi_{24}\rangle =|002021\rangle +|013132\rangle +|024243\rangle +|030304\rangle +|041410\rangle +|103200\rangle +|114311\rangle +|120422\rangle +|131033\rangle +|142144\rangle +|204434\rangle +|210040\rangle +|221101\rangle +|232212\rangle +|243323\rangle +|300113\rangle +|311224\rangle +|322330\rangle +|333441\rangle +|344002\rangle +|401342\rangle +|412403\rangle +|423014\rangle +|434120\rangle +|440231\rangle$,

$|\varphi_{25}\rangle =|003340\rangle +|014401\rangle +|020012\rangle +|031123\rangle +|042234\rangle +|104024\rangle +|110130\rangle +|121241\rangle +|132302\rangle +|143413\rangle +|200203\rangle +|211314\rangle +|222420\rangle +|233031\rangle +|244142\rangle +|301432\rangle +|312043\rangle +|323104\rangle +|334210\rangle +|340321\rangle +|402111\rangle +|413222\rangle +|424333\rangle +|430444\rangle +|441000\rangle$.

(II) The optimal code ${\left(\left(7,7^{3},3\right)\right)}_{7}$ constructed in Theorem 4.

Let $L_{0}$ be the following OA(49,7,7,2) and *M* be the following 343×7 matrix. Then $L_{i}=L_{0}⊕M\left(i\right)$ is an OA(49,7,7,2) where M(i) is the *i*-th row of *M* for i=1,2,…,343. Then, an $OA(7^{5},7,7,5)$ with MD=3 constructed by Lemma 3 has an orthogonal partition $\left\{L_{1},L_{2},\ldots ,L_{343}\right\}$. Every row of $L_{i}$ is put in kets and summed to produce a 2-uniform state $|\varphi_{i}\rangle$ for i=1,2,…,343. These states form an orthogonal basis of a subspace *Q* of ${C^{7}}^{⊗7}$. It follows from Theorem 1, where *Q* is the ${\left(\left(7,7^{3},3\right)\right)}_{7}$ QECC.

$$L_{0}^{T}=\left(\begin{array}{ccccccccccccccccccccccccccccccccccccccccccccccccc} 0 & 0 & 0 & 0 & 0 & 0 & 0 & 1 & 1 & 1 & 1 & 1 & 1 & 1 & 2 & 2 & 2 & 2 & 2 & 2 & 2 & 3 & 3 & 3 & 3 & 3 & 3 & 3 & 4 & 4 & 4 & 4 & 4 & 4 & 4 & 5 & 5 & 5 & 5 & 5 & 5 & 5 & 6 & 6 & 6 & 6 & 6 & 6 & 6\\ 0 & 1 & 2 & 3 & 4 & 5 & 6 & 0 & 1 & 2 & 3 & 4 & 5 & 6 & 0 & 1 & 2 & 3 & 4 & 5 & 6 & 0 & 1 & 2 & 3 & 4 & 5 & 6 & 0 & 1 & 2 & 3 & 4 & 5 & 6 & 0 & 1 & 2 & 3 & 4 & 5 & 6 & 0 & 1 & 2 & 3 & 4 & 5 & 6\\ 0 & 1 & 2 & 3 & 4 & 5 & 6 & 1 & 2 & 3 & 4 & 5 & 6 & 0 & 2 & 3 & 4 & 5 & 6 & 0 & 1 & 3 & 4 & 5 & 6 & 0 & 1 & 2 & 4 & 5 & 6 & 0 & 1 & 2 & 3 & 5 & 6 & 0 & 1 & 2 & 3 & 4 & 6 & 0 & 1 & 2 & 3 & 4 & 5\\ 0 & 1 & 2 & 3 & 4 & 5 & 6 & 2 & 3 & 4 & 5 & 6 & 0 & 1 & 4 & 5 & 6 & 0 & 1 & 2 & 3 & 6 & 0 & 1 & 2 & 3 & 4 & 5 & 1 & 2 & 3 & 4 & 5 & 6 & 0 & 3 & 4 & 5 & 6 & 0 & 1 & 2 & 5 & 6 & 0 & 1 & 2 & 3 & 4\\ 0 & 1 & 2 & 3 & 4 & 5 & 6 & 3 & 4 & 5 & 6 & 0 & 1 & 2 & 6 & 0 & 1 & 2 & 3 & 4 & 5 & 2 & 3 & 4 & 5 & 6 & 0 & 1 & 5 & 6 & 0 & 1 & 2 & 3 & 4 & 1 & 2 & 3 & 4 & 5 & 6 & 0 & 4 & 5 & 6 & 0 & 1 & 2 & 3\\ 0 & 1 & 2 & 3 & 4 & 5 & 6 & 4 & 5 & 6 & 0 & 1 & 2 & 3 & 1 & 2 & 3 & 4 & 5 & 6 & 0 & 5 & 6 & 0 & 1 & 2 & 3 & 4 & 2 & 3 & 4 & 5 & 6 & 0 & 1 & 6 & 0 & 1 & 2 & 3 & 4 & 5 & 3 & 4 & 5 & 6 & 0 & 1 & 2\\ 0 & 1 & 2 & 3 & 4 & 5 & 6 & 5 & 6 & 0 & 1 & 2 & 3 & 4 & 3 & 4 & 5 & 6 & 0 & 1 & 2 & 1 & 2 & 3 & 4 & 5 & 6 & 0 & 6 & 0 & 1 & 2 & 3 & 4 & 5 & 4 & 5 & 6 & 0 & 1 & 2 & 3 & 2 & 3 & 4 & 5 & 6 & 0 & 1 \end{array}\right),$$

$\;\;M^{T}=\left(\begin{array}{ccccccccccccccccccccccccccccccccccccccccccccccccccccccccccccccccccccccccccccccccc} 0 & 0 & 0 & 0 & 0 & 0 & 0 & 0 & 0 & 0 & 0 & 0 & 0 & 0 & 0 & 0 & 0 & 0 & 0 & 0 & 0 & 0 & 0 & 0 & 0 & 0 & 0 & 0 & 0 & 0 & 0 & 0 & 0 & 0 & 0 & 0 & 0 & 0 & 0 & 0 & 0 & 0 & 0 & 0 & 0 & 0 & 0 & 0 & 0 & 0 & 0 & 0 & 0 & 0 & 0 & 0 & 0 & 0 & 0 & 0 & 0 & 0 & 0 & 0 & 0 & 0 & 0 & 0 & 0 & 0 & 0 & 0 & 0 & 0 & 0 & 0 & 0 & 0 & 0 & 0 & 0\\ 0 & 0 & 0 & 0 & 0 & 0 & 0 & 0 & 0 & 0 & 0 & 0 & 0 & 0 & 0 & 0 & 0 & 0 & 0 & 0 & 0 & 0 & 0 & 0 & 0 & 0 & 0 & 0 & 0 & 0 & 0 & 0 & 0 & 0 & 0 & 0 & 0 & 0 & 0 & 0 & 0 & 0 & 0 & 0 & 0 & 0 & 0 & 0 & 0 & 0 & 0 & 0 & 0 & 0 & 0 & 0 & 0 & 0 & 0 & 0 & 0 & 0 & 0 & 0 & 0 & 0 & 0 & 0 & 0 & 0 & 0 & 0 & 0 & 0 & 0 & 0 & 0 & 0 & 0 & 0 & 0\\ 0 & 0 & 0 & 0 & 0 & 0 & 0 & 0 & 0 & 0 & 0 & 0 & 0 & 0 & 0 & 0 & 0 & 0 & 0 & 0 & 0 & 0 & 0 & 0 & 0 & 0 & 0 & 0 & 0 & 0 & 0 & 0 & 0 & 0 & 0 & 0 & 0 & 0 & 0 & 0 & 0 & 0 & 0 & 0 & 0 & 0 & 0 & 0 & 0 & 1 & 1 & 1 & 1 & 1 & 1 & 1 & 1 & 1 & 1 & 1 & 1 & 1 & 1 & 1 & 1 & 1 & 1 & 1 & 1 & 1 & 1 & 1 & 1 & 1 & 1 & 1 & 1 & 1 & 1 & 1 & 1\\ 0 & 0 & 0 & 0 & 0 & 0 & 0 & 1 & 1 & 1 & 1 & 1 & 1 & 1 & 2 & 2 & 2 & 2 & 2 & 2 & 2 & 3 & 3 & 3 & 3 & 3 & 3 & 3 & 4 & 4 & 4 & 4 & 4 & 4 & 4 & 5 & 5 & 5 & 5 & 5 & 5 & 5 & 6 & 6 & 6 & 6 & 6 & 6 & 6 & 0 & 0 & 0 & 0 & 0 & 0 & 0 & 1 & 1 & 1 & 1 & 1 & 1 & 1 & 2 & 2 & 2 & 2 & 2 & 2 & 2 & 3 & 3 & 3 & 3 & 3 & 3 & 3 & 4 & 4 & 4 & 4\\ 0 & 1 & 2 & 3 & 4 & 5 & 6 & 0 & 1 & 2 & 3 & 4 & 5 & 6 & 0 & 1 & 2 & 3 & 4 & 5 & 6 & 0 & 1 & 2 & 3 & 4 & 5 & 6 & 0 & 1 & 2 & 3 & 4 & 5 & 6 & 0 & 1 & 2 & 3 & 4 & 5 & 6 & 0 & 1 & 2 & 3 & 4 & 5 & 6 & 0 & 1 & 2 & 3 & 4 & 5 & 6 & 0 & 1 & 2 & 3 & 4 & 5 & 6 & 0 & 1 & 2 & 3 & 4 & 5 & 6 & 0 & 1 & 2 & 3 & 4 & 5 & 6 & 0 & 1 & 2 & 3\\ 0 & 3 & 6 & 2 & 5 & 1 & 4 & 3 & 6 & 2 & 5 & 1 & 4 & 0 & 6 & 2 & 5 & 1 & 4 & 0 & 3 & 2 & 5 & 1 & 4 & 0 & 3 & 6 & 5 & 1 & 4 & 0 & 3 & 6 & 2 & 1 & 4 & 0 & 3 & 6 & 2 & 5 & 4 & 0 & 3 & 6 & 2 & 5 & 1 & 1 & 4 & 0 & 3 & 6 & 2 & 5 & 4 & 0 & 3 & 6 & 2 & 5 & 1 & 0 & 3 & 6 & 2 & 5 & 1 & 4 & 3 & 6 & 2 & 5 & 1 & 4 & 0 & 6 & 2 & 5 & 1\\ 0 & 6 & 5 & 4 & 3 & 2 & 1 & 4 & 3 & 2 & 1 & 0 & 6 & 5 & 1 & 0 & 6 & 5 & 4 & 3 & 2 & 5 & 4 & 3 & 2 & 1 & 0 & 6 & 2 & 1 & 0 & 6 & 5 & 4 & 3 & 6 & 5 & 4 & 3 & 2 & 1 & 0 & 3 & 2 & 1 & 0 & 6 & 5 & 4 & 1 & 0 & 6 & 5 & 4 & 3 & 2 & 5 & 4 & 3 & 2 & 1 & 0 & 6 & 2 & 1 & 0 & 6 & 5 & 4 & 3 & 6 & 5 & 4 & 3 & 2 & 1 & 0 & 3 & 2 & 1 & 0 \end{array}\right.$

$\;\;\;\;\;\;\;\;\;\;\begin{array}{ccccccccccccccccccccccccccccccccccccccccccccccccccccccccccccccccccccccccccccccccc} 0 & 0 & 0 & 0 & 0 & 0 & 0 & 0 & 0 & 0 & 0 & 0 & 0 & 0 & 0 & 0 & 0 & 0 & 0 & 0 & 0 & 0 & 0 & 0 & 0 & 0 & 0 & 0 & 0 & 0 & 0 & 0 & 0 & 0 & 0 & 0 & 0 & 0 & 0 & 0 & 0 & 0 & 0 & 0 & 0 & 0 & 0 & 0 & 0 & 0 & 0 & 0 & 0 & 0 & 0 & 0 & 0 & 0 & 0 & 0 & 0 & 0 & 0 & 0 & 0 & 0 & 0 & 0 & 0 & 0 & 0 & 0 & 0 & 0 & 0 & 0 & 0 & 0 & 0 & 0 & 0\\ 0 & 0 & 0 & 0 & 0 & 0 & 0 & 0 & 0 & 0 & 0 & 0 & 0 & 0 & 0 & 0 & 0 & 0 & 0 & 0 & 0 & 0 & 0 & 0 & 0 & 0 & 0 & 0 & 0 & 0 & 0 & 0 & 0 & 0 & 0 & 0 & 0 & 0 & 0 & 0 & 0 & 0 & 0 & 0 & 0 & 0 & 0 & 0 & 0 & 0 & 0 & 0 & 0 & 0 & 0 & 0 & 0 & 0 & 0 & 0 & 0 & 0 & 0 & 0 & 0 & 0 & 0 & 0 & 0 & 0 & 0 & 0 & 0 & 0 & 0 & 0 & 0 & 0 & 0 & 0 & 0\\ 1 & 1 & 1 & 1 & 1 & 1 & 1 & 1 & 1 & 1 & 1 & 1 & 1 & 1 & 1 & 1 & 1 & 2 & 2 & 2 & 2 & 2 & 2 & 2 & 2 & 2 & 2 & 2 & 2 & 2 & 2 & 2 & 2 & 2 & 2 & 2 & 2 & 2 & 2 & 2 & 2 & 2 & 2 & 2 & 2 & 2 & 2 & 2 & 2 & 2 & 2 & 2 & 2 & 2 & 2 & 2 & 2 & 2 & 2 & 2 & 2 & 2 & 2 & 2 & 2 & 2 & 3 & 3 & 3 & 3 & 3 & 3 & 3 & 3 & 3 & 3 & 3 & 3 & 3 & 3 & 3\\ 4 & 4 & 4 & 5 & 5 & 5 & 5 & 5 & 5 & 5 & 6 & 6 & 6 & 6 & 6 & 6 & 6 & 0 & 0 & 0 & 0 & 0 & 0 & 0 & 1 & 1 & 1 & 1 & 1 & 1 & 1 & 2 & 2 & 2 & 2 & 2 & 2 & 2 & 3 & 3 & 3 & 3 & 3 & 3 & 3 & 4 & 4 & 4 & 4 & 4 & 4 & 4 & 5 & 5 & 5 & 5 & 5 & 5 & 5 & 6 & 6 & 6 & 6 & 6 & 6 & 6 & 0 & 0 & 0 & 0 & 0 & 0 & 0 & 1 & 1 & 1 & 1 & 1 & 1 & 1 & 2\\ 4 & 5 & 6 & 0 & 1 & 2 & 3 & 4 & 5 & 6 & 0 & 1 & 2 & 3 & 4 & 5 & 6 & 0 & 1 & 2 & 3 & 4 & 5 & 6 & 0 & 1 & 2 & 3 & 4 & 5 & 6 & 0 & 1 & 2 & 3 & 4 & 5 & 6 & 0 & 1 & 2 & 3 & 4 & 5 & 6 & 0 & 1 & 2 & 3 & 4 & 5 & 6 & 0 & 1 & 2 & 3 & 4 & 5 & 6 & 0 & 1 & 2 & 3 & 4 & 5 & 6 & 0 & 1 & 2 & 3 & 4 & 5 & 6 & 0 & 1 & 2 & 3 & 4 & 5 & 6 & 0\\ 4 & 0 & 3 & 2 & 5 & 1 & 4 & 0 & 3 & 6 & 5 & 1 & 4 & 0 & 3 & 6 & 2 & 2 & 5 & 1 & 4 & 0 & 3 & 6 & 5 & 1 & 4 & 0 & 3 & 6 & 2 & 1 & 4 & 0 & 3 & 6 & 2 & 5 & 4 & 0 & 3 & 6 & 2 & 5 & 1 & 0 & 3 & 6 & 2 & 5 & 1 & 4 & 3 & 6 & 2 & 5 & 1 & 4 & 0 & 6 & 2 & 5 & 1 & 4 & 0 & 3 & 3 & 6 & 2 & 5 & 1 & 4 & 0 & 6 & 2 & 5 & 1 & 4 & 0 & 3 & 2\\ 6 & 5 & 4 & 0 & 6 & 5 & 4 & 3 & 2 & 1 & 4 & 3 & 2 & 1 & 0 & 6 & 5 & 2 & 1 & 0 & 6 & 5 & 4 & 3 & 6 & 5 & 4 & 3 & 2 & 1 & 0 & 3 & 2 & 1 & 0 & 6 & 5 & 4 & 0 & 6 & 5 & 4 & 3 & 2 & 1 & 4 & 3 & 2 & 1 & 0 & 6 & 5 & 1 & 0 & 6 & 5 & 4 & 3 & 2 & 5 & 4 & 3 & 2 & 1 & 0 & 6 & 3 & 2 & 1 & 0 & 6 & 5 & 4 & 0 & 6 & 5 & 4 & 3 & 2 & 1 & 4 \end{array}$

$\;\;\;\;\;\;\;\;\;\;\begin{array}{ccccccccccccccccccccccccccccccccccccccccccccccccccccccccccccccccccccccccccccccccc} 0 & 0 & 0 & 0 & 0 & 0 & 0 & 0 & 0 & 0 & 0 & 0 & 0 & 0 & 0 & 0 & 0 & 0 & 0 & 0 & 0 & 0 & 0 & 0 & 0 & 0 & 0 & 0 & 0 & 0 & 0 & 0 & 0 & 0 & 0 & 0 & 0 & 0 & 0 & 0 & 0 & 0 & 0 & 0 & 0 & 0 & 0 & 0 & 0 & 0 & 0 & 0 & 0 & 0 & 0 & 0 & 0 & 0 & 0 & 0 & 0 & 0 & 0 & 0 & 0 & 0 & 0 & 0 & 0 & 0 & 0 & 0 & 0 & 0 & 0 & 0 & 0 & 0 & 0 & 0 & 0\\ 0 & 0 & 0 & 0 & 0 & 0 & 0 & 0 & 0 & 0 & 0 & 0 & 0 & 0 & 0 & 0 & 0 & 0 & 0 & 0 & 0 & 0 & 0 & 0 & 0 & 0 & 0 & 0 & 0 & 0 & 0 & 0 & 0 & 0 & 0 & 0 & 0 & 0 & 0 & 0 & 0 & 0 & 0 & 0 & 0 & 0 & 0 & 0 & 0 & 0 & 0 & 0 & 0 & 0 & 0 & 0 & 0 & 0 & 0 & 0 & 0 & 0 & 0 & 0 & 0 & 0 & 0 & 0 & 0 & 0 & 0 & 0 & 0 & 0 & 0 & 0 & 0 & 0 & 0 & 0 & 0\\ 3 & 3 & 3 & 3 & 3 & 3 & 3 & 3 & 3 & 3 & 3 & 3 & 3 & 3 & 3 & 3 & 3 & 3 & 3 & 3 & 3 & 3 & 3 & 3 & 3 & 3 & 3 & 3 & 3 & 3 & 3 & 3 & 3 & 3 & 4 & 4 & 4 & 4 & 4 & 4 & 4 & 4 & 4 & 4 & 4 & 4 & 4 & 4 & 4 & 4 & 4 & 4 & 4 & 4 & 4 & 4 & 4 & 4 & 4 & 4 & 4 & 4 & 4 & 4 & 4 & 4 & 4 & 4 & 4 & 4 & 4 & 4 & 4 & 4 & 4 & 4 & 4 & 4 & 4 & 4 & 4\\ 2 & 2 & 2 & 2 & 2 & 2 & 3 & 3 & 3 & 3 & 3 & 3 & 3 & 4 & 4 & 4 & 4 & 4 & 4 & 4 & 5 & 5 & 5 & 5 & 5 & 5 & 5 & 6 & 6 & 6 & 6 & 6 & 6 & 6 & 0 & 0 & 0 & 0 & 0 & 0 & 0 & 1 & 1 & 1 & 1 & 1 & 1 & 1 & 2 & 2 & 2 & 2 & 2 & 2 & 2 & 3 & 3 & 3 & 3 & 3 & 3 & 3 & 4 & 4 & 4 & 4 & 4 & 4 & 4 & 5 & 5 & 5 & 5 & 5 & 5 & 5 & 6 & 6 & 6 & 6 & 6\\ 1 & 2 & 3 & 4 & 5 & 6 & 0 & 1 & 2 & 3 & 4 & 5 & 6 & 0 & 1 & 2 & 3 & 4 & 5 & 6 & 0 & 1 & 2 & 3 & 4 & 5 & 6 & 0 & 1 & 2 & 3 & 4 & 5 & 6 & 0 & 1 & 2 & 3 & 4 & 5 & 6 & 0 & 1 & 2 & 3 & 4 & 5 & 6 & 0 & 1 & 2 & 3 & 4 & 5 & 6 & 0 & 1 & 2 & 3 & 4 & 5 & 6 & 0 & 1 & 2 & 3 & 4 & 5 & 6 & 0 & 1 & 2 & 3 & 4 & 5 & 6 & 0 & 1 & 2 & 3 & 4\\ 5 & 1 & 4 & 0 & 3 & 6 & 5 & 1 & 4 & 0 & 3 & 6 & 2 & 1 & 4 & 0 & 3 & 6 & 2 & 5 & 4 & 0 & 3 & 6 & 2 & 5 & 1 & 0 & 3 & 6 & 2 & 5 & 1 & 4 & 4 & 0 & 3 & 6 & 2 & 5 & 1 & 0 & 3 & 6 & 2 & 5 & 1 & 4 & 3 & 6 & 2 & 5 & 1 & 4 & 0 & 6 & 2 & 5 & 1 & 4 & 0 & 3 & 2 & 5 & 1 & 4 & 0 & 3 & 6 & 5 & 1 & 4 & 0 & 3 & 6 & 2 & 1 & 4 & 0 & 3 & 6\\ 3 & 2 & 1 & 0 & 6 & 5 & 1 & 0 & 6 & 5 & 4 & 3 & 2 & 5 & 4 & 3 & 2 & 1 & 0 & 6 & 2 & 1 & 0 & 6 & 5 & 4 & 3 & 6 & 5 & 4 & 3 & 2 & 1 & 0 & 4 & 3 & 2 & 1 & 0 & 6 & 5 & 1 & 0 & 6 & 5 & 4 & 3 & 2 & 5 & 4 & 3 & 2 & 1 & 0 & 6 & 2 & 1 & 0 & 6 & 5 & 4 & 3 & 6 & 5 & 4 & 3 & 2 & 1 & 0 & 3 & 2 & 1 & 0 & 6 & 5 & 4 & 0 & 6 & 5 & 4 & 3 \end{array}$

$\;\;\;\;\;\;\;\;\;\;\begin{array}{ccccccccccccccccccccccccccccccccccccccccccccccccccccccccccccccccccccccccccccccccc} 0 & 0 & 0 & 0 & 0 & 0 & 0 & 0 & 0 & 0 & 0 & 0 & 0 & 0 & 0 & 0 & 0 & 0 & 0 & 0 & 0 & 0 & 0 & 0 & 0 & 0 & 0 & 0 & 0 & 0 & 0 & 0 & 0 & 0 & 0 & 0 & 0 & 0 & 0 & 0 & 0 & 0 & 0 & 0 & 0 & 0 & 0 & 0 & 0 & 0 & 0 & 0 & 0 & 0 & 0 & 0 & 0 & 0 & 0 & 0 & 0 & 0 & 0 & 0 & 0 & 0 & 0 & 0 & 0 & 0 & 0 & 0 & 0 & 0 & 0 & 0 & 0 & 0 & 0 & 0 & 0\\ 0 & 0 & 0 & 0 & 0 & 0 & 0 & 0 & 0 & 0 & 0 & 0 & 0 & 0 & 0 & 0 & 0 & 0 & 0 & 0 & 0 & 0 & 0 & 0 & 0 & 0 & 0 & 0 & 0 & 0 & 0 & 0 & 0 & 0 & 0 & 0 & 0 & 0 & 0 & 0 & 0 & 0 & 0 & 0 & 0 & 0 & 0 & 0 & 0 & 0 & 0 & 0 & 0 & 0 & 0 & 0 & 0 & 0 & 0 & 0 & 0 & 0 & 0 & 0 & 0 & 0 & 0 & 0 & 0 & 0 & 0 & 0 & 0 & 0 & 0 & 0 & 0 & 0 & 0 & 0 & 0\\ 4 & 4 & 5 & 5 & 5 & 5 & 5 & 5 & 5 & 5 & 5 & 5 & 5 & 5 & 5 & 5 & 5 & 5 & 5 & 5 & 5 & 5 & 5 & 5 & 5 & 5 & 5 & 5 & 5 & 5 & 5 & 5 & 5 & 5 & 5 & 5 & 5 & 5 & 5 & 5 & 5 & 5 & 5 & 5 & 5 & 5 & 5 & 5 & 5 & 5 & 5 & 6 & 6 & 6 & 6 & 6 & 6 & 6 & 6 & 6 & 6 & 6 & 6 & 6 & 6 & 6 & 6 & 6 & 6 & 6 & 6 & 6 & 6 & 6 & 6 & 6 & 6 & 6 & 6 & 6 & 6\\ 6 & 6 & 0 & 0 & 0 & 0 & 0 & 0 & 0 & 1 & 1 & 1 & 1 & 1 & 1 & 1 & 2 & 2 & 2 & 2 & 2 & 2 & 2 & 3 & 3 & 3 & 3 & 3 & 3 & 3 & 4 & 4 & 4 & 4 & 4 & 4 & 4 & 5 & 5 & 5 & 5 & 5 & 5 & 5 & 6 & 6 & 6 & 6 & 6 & 6 & 6 & 0 & 0 & 0 & 0 & 0 & 0 & 0 & 1 & 1 & 1 & 1 & 1 & 1 & 1 & 2 & 2 & 2 & 2 & 2 & 2 & 2 & 3 & 3 & 3 & 3 & 3 & 3 & 3 & 4 & 4\\ 5 & 6 & 0 & 1 & 2 & 3 & 4 & 5 & 6 & 0 & 1 & 2 & 3 & 4 & 5 & 6 & 0 & 1 & 2 & 3 & 4 & 5 & 6 & 0 & 1 & 2 & 3 & 4 & 5 & 6 & 0 & 1 & 2 & 3 & 4 & 5 & 6 & 0 & 1 & 2 & 3 & 4 & 5 & 6 & 0 & 1 & 2 & 3 & 4 & 5 & 6 & 0 & 1 & 2 & 3 & 4 & 5 & 6 & 0 & 1 & 2 & 3 & 4 & 5 & 6 & 0 & 1 & 2 & 3 & 4 & 5 & 6 & 0 & 1 & 2 & 3 & 4 & 5 & 6 & 0 & 1\\ 2 & 5 & 5 & 1 & 4 & 0 & 3 & 6 & 2 & 1 & 4 & 0 & 3 & 6 & 2 & 5 & 4 & 0 & 3 & 6 & 2 & 5 & 1 & 0 & 3 & 6 & 2 & 5 & 1 & 4 & 3 & 6 & 2 & 5 & 1 & 4 & 0 & 6 & 2 & 5 & 1 & 4 & 0 & 3 & 2 & 5 & 1 & 4 & 0 & 3 & 6 & 6 & 2 & 5 & 1 & 4 & 0 & 3 & 2 & 5 & 1 & 4 & 0 & 3 & 6 & 5 & 1 & 4 & 0 & 3 & 6 & 2 & 1 & 4 & 0 & 3 & 6 & 2 & 5 & 4 & 0\\ 2 & 1 & 5 & 4 & 3 & 2 & 1 & 0 & 6 & 2 & 1 & 0 & 6 & 5 & 4 & 3 & 6 & 5 & 4 & 3 & 2 & 1 & 0 & 3 & 2 & 1 & 0 & 6 & 5 & 4 & 0 & 6 & 5 & 4 & 3 & 2 & 1 & 4 & 3 & 2 & 1 & 0 & 6 & 5 & 1 & 0 & 6 & 5 & 4 & 3 & 2 & 6 & 5 & 4 & 3 & 2 & 1 & 0 & 3 & 2 & 1 & 0 & 6 & 5 & 4 & 0 & 6 & 5 & 4 & 3 & 2 & 1 & 4 & 3 & 2 & 1 & 0 & 6 & 5 & 1 & 0 \end{array}$

$\;\;\;\;\;\;\;\left.\begin{array}{ccccccccccccccccccc} 0 & 0 & 0 & 0 & 0 & 0 & 0 & 0 & 0 & 0 & 0 & 0 & 0 & 0 & 0 & 0 & 0 & 0 & 0\\ 0 & 0 & 0 & 0 & 0 & 0 & 0 & 0 & 0 & 0 & 0 & 0 & 0 & 0 & 0 & 0 & 0 & 0 & 0\\ 6 & 6 & 6 & 6 & 6 & 6 & 6 & 6 & 6 & 6 & 6 & 6 & 6 & 6 & 6 & 6 & 6 & 6 & 6\\ 4 & 4 & 4 & 4 & 4 & 5 & 5 & 5 & 5 & 5 & 5 & 5 & 6 & 6 & 6 & 6 & 6 & 6 & 6\\ 2 & 3 & 4 & 5 & 6 & 0 & 1 & 2 & 3 & 4 & 5 & 6 & 0 & 1 & 2 & 3 & 4 & 5 & 6\\ 3 & 6 & 2 & 5 & 1 & 0 & 3 & 6 & 2 & 5 & 1 & 4 & 3 & 6 & 2 & 5 & 1 & 4 & 0\\ 6 & 5 & 4 & 3 & 2 & 5 & 4 & 3 & 2 & 1 & 0 & 6 & 2 & 1 & 0 & 6 & 5 & 4 & 3 \end{array}\right).$
